# Stolen Lives: Redress for Slavery’s and Jim Crow’s Ongoing Theft of Lifespan

**DOI:** 10.7758/rsf.2024.10.2.04

**Published:** 2024-06

**Authors:** ELIZABETH WRIGLEY-FIELD

**Affiliations:** Department of Sociology and Minnesota Population Center at the University of Minnesota, Twin Cities, United States.

**Keywords:** lifespan disparities, time, freedom, health, wealth

## Abstract

Reparations proposals typically target wealth. Yet slavery’s and Jim Crow’s long echoes also steal time, such as by producing shorter Black lifespans even today. I argue that lost time should be considered an independent target for redress; identify challenges to doing so; and provide examples of what reparations redressing lost lifespan could look like. To identify quantitative targets for redress, I analyze area-level relationships between Black lifespans and six measures of intensity of slavery, Jim Crow, and racial terror. Results reveal inconsistent relationships across measures, suggesting difficulties in grounding a target for redress in such variation. Instead, I propose that policies aim to redress the national lifespan gap between White and Black Americans. The article concludes with a typology of potential strategies for such redress.

Among the stories of violence Margaret Burnham recounts in *By Hands Now Known: Jim Crow’s Legal Executioners* is a series of incidents in Alabama’s Union Springs in 1945 (Burnham 2022, 136–39). A stretch of Black businesses included a shop and cafe owned by Edgar Bernard Thomas and, next door, a barbershop owned by Reverend James L. Pinckney. On October 13, Thomas’s business was visited by two police officers. One was Dewey Columbus Bradley, who had already threatened Thomas (perhaps over interest in a woman with whom Thomas was romantically linked). The other was the assistant police chief, Hollis Eugene Whittle, who came armed with a sawed-off shotgun. In front of witnesses, Officer Bradley shot Thomas in the face and shot him several more times as he fled, killing the sixty-three-year-old. From his adjacent business, Reverend Pinckney heard Bradley tell Thomas, “We’re going to run this damn town. I’ll kill every black son of a bitch in the street.” Later that day, Pinckney was warned by the chief of police that he must leave town to avoid Thomas’s fate. He listened, fleeing to the woods (and ultimately to Chicago) and leaving his wife and his business behind.

The tragedies and injustices enacted on Thomas and Pinckney on this one day in October 1945 illustrate a core aspect of Jim Crow: the intertwining of theft and violence. Pinckney lost his business and his home; Thomas lost his life. Among the consequences of the widespread racial terror recounted in Burnham’s extensively documented history are an immense loss of wealth among Black Americans who had sought to amass security through businesses and property, and the losses of life that facilitated many of those thefts. Some victims of racist murder, faced with untenable choices, chose dignity and defiance over life; or at least, over preserving their lives at any cost. Others were murdered with no such choice. As Burnham puts it when summarizing the context of resistance to Jim Crow elsewhere during the same period, “In Birmingham in the 1940s, a Black person—any Black person—could have been killed by a white person—any white person. And thus every Black person had to make peace with the burden and duty of resistance, reckon with premature death, determine their personal point of no return, and countenance the politics of Black revolt, whether or not they wanted to” (Burnham 2022, 190–91). The threat of lost life was foundational to Jim Crow’s terror and to its terrible edifice.

These linked thefts—of wealth and of life—are twin faces of racial domination in the United States. They were the foundations of enslavement, which built extraordinary wealth through stolen labor power: life’s work (Marx [1867] 1981, 914–26). As the historian James Oakes (2015) puts it:

When abolitionists denounced slavery as “theft,” they had two different kinds of robbery in mind. One was the day-by-day, year-by-year, theft of the fruits of the slave’s labor. But they were also thinking of a different, more fundamental kind of theft. Human beings own themselves, as a natural right, a right of property, abolitionists argued. So when masters claimed slaves as their own they were effectively robbing the slaves of their property in themselves.

Oakes develops the concept of *property in themselves* to make a specific point about the complex relationship between chattel slavery and capitalism. But his distinction is also useful from a broader perspective: slavery entailed not only the theft of the fruits of the labor of enslaved people, but also the theft of their self-determination. The starting point of this article is this reminder to think broadly about self-determination as the most fundamental theft enabled and enacted by America’s systems of racial domination.^1^

Scholars and activists have articulated proposals for reparations to address this history, with increasing specificity and, over the past decade, receiving increasing attention. These proposals vary in their details, whether payments to individual African descendants of slaves (Darity and Mullen 2020), community land trusts (Franke 2019), or other mechanisms. Yet nearly all share one target for redress: stolen wealth. Whether focused on the present-day value of specific thefts and injustices associated with chattel slavery—estimated at some trillions of dollars even without accounting for Jim Crow apartheid (America 1990; Craemer 2015; Darity and Frank 2003)—or remediating the Black-White wealth gap broadly (Darity, Mullen, and Slaughter 2022), these proposals identify wealth as the appropriate mechanism for redress even when the goal is to improve health (Williams and Collins 2004; Outterson 2005; 2008; Bassett and Galea 2020; Taifa 2020; Richardson et al. 2021; Lawrenz 2022; Thakur and Martinez 2023).^2^ If stolen wealth and stolen time are the two sides of America’s system of racial domination, discussions of reparations for that system have, by and large, focused squarely on one side alone.

On one level, this focus makes sense. Wealth is, perhaps, the ultimate proxy for power; and research suggests that Black families’ economic circumstances today are still powerfully shaped by their own families’ direct exposure to enslavement and Jim Crow (Althoff and Reichardt, n.d.),^3^ as well as being powerfully shaped by the larger economic context that those institutions created. Yet it is not obvious that attempts to redress stolen wealth can adequately redress the other face of racial domination: stolen time. The loss of lifespan associated with being Black in the United States is shocking in its magnitude. Black mortality in the United States every single year has been higher even than White mortality was during the first year of the COVID-19 pandemic (Wrigley-Field 2020).^4^ Even before the pandemic, about sixty-eight thousand more Black Americans died each year than would if the Black population had the White population’s death rates (author’s calculation). As the literary historian Saidiya Hartman puts it, “Racism is a distribution of death, controlled depletion, and a brutal allocation of chances at life” (Rodriques 2022).

If wealth is the best proxy for power, then, I claim, time may be the best proxy for freedom. This claim builds on a long theoretical tradition (Goodin 2008; Konzcal 2021; Cohen, n.d.; 2018; Rose 2016, 66–91; Marx [1867] 1981, 340–416; Downs 2012, 16) and also a practical one, in the sense that—according to the analysis of historian Jim Downs—“health formed a central part of freedpeople’s campaign for rights” (Downs 2012, 166).^5^ Understanding stolen time through this lens returns the theft of time from a solely economistic understanding (time spent producing wealth) to a human footing (time as the fundamental resource through which we pursue our own most important goals). Lost lifespan may be a somewhat reductive proxy for a broader construct: how lives, or whose lives, are valued. Yet as the starkest possible determinant of how much time each of us gets, it is also an intrinsically meaningful outcome.

Because wealth is produced by human beings’ effortful time, there is no sharp distinction between slavery as stolen wealth and slavery as stolen time. This insight is particularly clear in work by Thomas Craemer (2015), which estimates the wealth stolen through slavery as the wages that were denied to enslaved people for their long hours and years of work, yielding estimates of about $6 to $14 trillion in total, depending on whether the stolen time in question is the time spent working or the time spent under the control of the enslaver, that is, all time. This calculation hinges on enslaved people’s time as the *proximate* object of theft and translates that time into wealth as the ultimate metric for redress.^6^ Here, however, I explore treating time as the *ultimate* object of theft and ask, among other questions, whether wealth transfers must—and can—serve as a proximate means of addressing this theft. That is, I explore the idea that articulating stolen time itself, as distinct from stolen wealth, as a target for redress might also suggest additional avenues for repair that need not—and perhaps cannot—be fulfilled via wealth transfers.

Can lifespans cut short due to racial subjugation truly be redressed? Certainly, no form of redress is possible to those whose life has already been taken—a point I return to at the end of this article. But two other potential targets for redress remain: redress to those who have lost time with kin due to (relatively recent) violent racial subjugation, and redress for ongoing disparities in length of life that are associated with slavery and Jim Crow. For kin (formal or chosen), the particular psychosocial harms that follow from a loved one’s life being unjustly cut short could be compounded by the inability to publicly mourn—a form of “disenfranchised grief” (Doka 1999). The stories collected by Burnham (2022) reflect, over and over again, that mourning the victims of Jim Crow violence risked further violence.^7^ However, I focus here on redress for ongoing inequities in lifespan linked to America’s history of enslavement and Jim Crow.

In what follows, I consider two questions. First, can the contemporary loss of lifetime associated with slavery and Jim Crow be quantified? Second, what avenues are there to redress stolen time? In the vein of the second question, I ask: to what extent can wealth-based reparations provide redress for these losses, and what else might provide redress? In general, this article advances an argument that reparations can better address the scale of harm committed by American slavery and Jim Crow if they incorporate lifespan, while providing a framework for understanding key difficulties and choices in doing so in practice.

## CAN THE TWENTY-FIRST-CENTURY LIFESPAN COST OF SLAVERY AND JIM CROW BE QUANTIFIED?

If one wants to identify quantitative targets for redress, there are two natural of proceeding. One is to use the full White-B lack lifespan disparity as a target; the other is to compare states with different intensity of histories of enslavement, Jim Crow, and racial terror. Here I ask whether this second, more specific strategy can outperform the first, blunter approach in giving a meaningful quantitative target for redress.

Specifically, I explore how state-level variation in Black lifespans and in the White-Black lifespan gap is associated with states’ enslavement and Jim Crow histories and analyze what the results imply about the ability to identify meaningful measures for quantifying the cost of those institutions in lifespans today. I also analyze those histories’ relationship with White lifespans, insofar as this helps to understand how those histories structure within-state White-Black lifespan gaps. Many measures of enslavement, Jim Crow, and racial terror are possible. Here I show that these measures do not agree, even qualitatively, on which states had the greatest intensity of these atrocities, nor on the relationship of that intensity to Black lifespan and White-Black lifespan disparities. These results imply that this natural method for identifying targets for redress may not work, at least, not without a strong basis for preferring a particular metric to others.

To contextualize the results to follow about state-level lifespan variation among the former slaveholding states, I provide some brief descriptive facts about those states as a whole (illustrated in the online appendix).^8^ Black and White lifespans are each lower in the states that had enslaved populations in 1860 than in other states, by, at the median, about two years for White lifespans and nearly three years for Black. In addition, Black lifespans are substantially lower than White lifespans in each group by, at the median, more than three years in the 1860 slaveholding states and nearly three years in the other states. These comparisons fail to capture the fact that the Black population disproportionately lives in the former enslaving states, which is why the total lifespan disparity in the United States is larger than the disparity in each of these regions: life expectancy in 2019 (before the COVID-19 pandemic) was 74.8 for non-Hispanic Black, and 78.8 for non-Hispanic White, populations.

In what follows, I analyze whether these lifespan outcomes also vary within the former enslaving states in association with a variety of measures of the intensity of slavery and Jim Crow practices in each state, in order to see whether there is a consistent relationship across measures. I do not use control variables because the point is to capture relationships between American racial domination and lifespan that flow through multifaceted pathways that would implicate almost any realistic confounder as also being an intermediary variable, and because the chance of a well-identified causal effect is slim in any case. Instead of attempting to causally isolate slavery and Jim Crow regimes as a cause net of some other variables (that they also no doubt contributed to causing), then, I explore whether their overall association with lifespans can help us conceptualize racial disparities in lifespan as a target for redress, much as total racial disparities in wealth have been identified as one plausible target (Darity, Mullen, and Slaughter 2022).

### Quantifying State-Level Variation: Data

The primary lifespan data are measures of state-specific period life expectancy for non-Hispanic Black and White Americans, calculated as part of the County Health Rankings and Roadmaps (CHRR) dataset (University of Wisconsin Population Health Institute, n.d.), based on restricted-access lifespan data from the National Vital Statistics System.^9^ Because the long-t erm consequences for mortality of the COVID-19 pandemic are still unknown, this article uses pre-pandemic lifespans by using the 2021 CHRR release, which draws data from 2017 to 2019. Life expectancy measures in this dataset therefore represent the average lifespan of a hypothetical birth cohort whose mortality conditions, over their entire lives, were those of the same-race 2017–2019 population in their state. I treat Washington, D.C., as a state.

I use state-level lifespans, rather than more disaggregated geographies such as counties, for two reasons: first, because it is not clear that counties have any analog of state governments’ “critical role in defining racial categories and policing their boundaries” (Bruch, Rosenthal, and Soss 2019, 164); second, because some county-level estimates from the CHRR seem clearly implausible (for example, life expectancy above one hundred for both White and Black populations) and likely reflect life expectancy’s sensitivity to small-area variation. However, in an additional analysis reported in the appendix, I use county-level lifespans from a different source, the Institute for Health Metrics and Evaluation (Dwyer-Lindgren et al. 2022), which raise some different methodological challenges.

I analyze two outcomes that each imply different benchmarks for conceptualizing the lifespan loss associated with states’ participation in slavery and Jim Crow. First, I analyze state-level variation in Black life expectancy. This outcome implicitly uses Black life expectancy in other states as a benchmark (or baseline). Second, I analyze state-level variation in the White-Black life expectancy gap. This outcome uses White people in the same state as a benchmark.

As independent variables, I use four distinct measurements of the intensity of slavery and Jim Crow at the state level. First, I examine the proportion of the state population that was enslaved in 1860. This measure was previously found to be associated, at the county level, with lower Black, and higher White, life expectancy, net of various control variables (Reece 2022). Second, I examine counts of Jim Crow laws passed by each state before 1950. These laws, some seven hundred in total, were originally collected by Pauli Murray ([1950] 2016) and were digitized and extended by Althoff and Reichardt (n.d.). Third, I examine the quality of segregated Black schools. The school quality measure was also constructed by Althoff and Reichardt, who find large long-term economic consequences of both Jim Crow laws and Jim Crow school quality, using data on teacher salaries, student-to-teacher ratios, and term lengths in Black segregated schools during the Jim Crow era, all previously collected by David Card and Alan Krueger (1992). I reverse-code this measure to indicate inadequate quality, so that, like the other measures, it reflects the intensity of racial oppression.

Fourth, I examine the historic racial regimes index (HRR) developed by Regina Baker (2022), a composite index of measures of slavery, sharecropping, disfranchisement, and segregation—measures collectively spanning the antebellum period, Reconstruction, and Jim Crow. Baker uses this scale to assess the legacies of racial regimes for poverty across southern states, finding that a stronger historic racial regime is associated with worse Black poverty and, especially, Black-White economic inequalities.^10^

Finally, in the appendix, I additionally explore two additional measures designed to capture the intensity of racial terror at the state and county levels, respectively. These are, first, counts of Jim Crow violent incidents, collected by the Burnham-Nobles Digital Archive (Civil Rights & Restorative Justice Project, n.d.); and second, counts of lynchings of Black Americans between 1883 and 1941, collected by Charles Seguin and David Rigby (2019).^11^ Both measures were collected from historical newspaper accounts, a source that raises distinctive measurement concerns, as described in the appendix, though these concerns are likely to be substantially greater for the broader measure of violence than for lynchings. Extrajudicial violence also bears a complex relationship with racial domination, in that it may occur most readily when other, more formalized and routinized means of domination are not in operation or not reliable (Troesken and Walsh 2019).^12^ Yet, despite these weaknesses and calls to more comprehensively measure racial violence, much evidence based on these and other data sources show long-term consequences of racial terror (Cunningham, Lee, and Ward 2021).

Each of these measures is defined for a distinct set of states, but their bivariate correlations, in the states where each respective pair exists, vary widely. Unsurprisingly given that it is constructed from other measures or analogs of them, the HRR is tightly correlated with most of the other measures: its correlation with (reverse-coded) Black school quality is 0.94, with the proportion enslaved in 1860 (which is one of its four components) is 0.89, and with the count of Jim Crow laws (a much simplified version of which is one of the HRR’s components) is 0.71.

### Quantifying State-Level Variation: Results

Results of these analyses are summarized in table 1, which presents, for each measure, the difference between each outcome at the 75th and the 25th percentile values of that measure (with singular outliers omitted, as noted). Detailed, graphical results and summaries are presented in the appendix. State-level measures that include Washington, D.C.—the proportion enslaved in 1860, the count of Jim Crow laws, and the quality of Jim Crow schools—are reported with and without that observation because it is an extreme outlier of White life expectancy among Jim Crow states, with the highest state-level White life expectancy in the entire United States. Washington, D.C., has the fourth-lowest Black life expectancy in the United States, which does not make it an outlier among Jim Crow states. Additionally, the count of Jim Crow laws is also reported without Louisiana, which passed ninety-eight such laws, nearly 50 percent more than the next-highest state, Virginia, which passed sixty-six, an intensity of legal disfranchisement that was extraordinarily effective and damaging (Keele, Cubbison, and White 2021). These two outliers substantially affect the bivariate relationships summarized in table 1 when they are included.

Across measures, the relationship of the intensity of slavery or Jim Crow—when comparing states that had slavery in 1860 or Jim Crow laws—to Black life expectancy and to the White-Black life expectancy gap is somewhat inconsistent. For Black life expectancy, the proportion enslaved in 1860, poor-quality Jim Crow schools, and the HRR index all have a negative relationship, and the count of Jim Crow laws has a positive relationship. For the White-Black life expectancy gap, the proportion enslaved in 1860 and the count of Jim Crow laws have an unexpected negative relationship (as do both measures of violence analyzed in the appendix); the extent of poor-quality Jim Crow schools has almost no relationship; and only the HRR index has the expected positive relationship, which is driven primarily by that index’s positive association with White life expectancy. In general, the geographic variation in the White-Black life expectancy gap analyzed here is more heavily driven by variation in White life expectancy than by variation in Black life expectancy.

The inconsistency of these results is perhaps surprising in light of research finding county-level negative associations (net of controls) between Black life expectancy and the proportion of the population enslaved in 1860 (Reece 2022).^13^ More broadly, the research on the “long arm of slavery” finds enduring spatial and place-based differences, based on county-level and state-level histories of enslavement, in Black levels of, and Black-White disparities in, outcomes such as poverty (O’Connell 2012; Baker 2022), educational attainment (Bertocchi and Dimico 2012), arrest rates (Ward 2022), and harsh prison sentences (Gottlieb and Flynn 2021). County-level lynching histories predict contemporary use of corporal punishment in public schools, particularly for Black students (Ward 2022). States’ and counties’ enslavement histories predict their consequential cultural and institutional features like the political attitudes of their White residents (Acharya, Blackwell, and Sen 2016), their contemporary political suppression of Black votes and the paucity of their welfare state provisions (Williams, Logan, and Hardy 2021), and the late-twentieth-century growth of their incarceration apparatuses (Duxbury 2023), though this latter association partly reflects a complex history in which other forms of labor control predominated in the South in the early twentieth century (Muller 2021). This large research program pinpointing slavery’s long-term space- and place-based legacies (reviewed in Cunningham, Lee, and Ward 2021) is not without nuance and qualification, such as explorations of how demographic change enables and constrains the persistence of these relationships (O’Connell, Curtis, and DeWaard 2020) and analyses of mid-century racial terror as an intermediary mechanism between slavery and contemporary homicide that varied in intensity (Petersen and Ward 2015). Yet despite its growing complexity, this research program’s overall message is clear: slavery reverberates. Its echoes are so vast and all-encompassing that they are best approached on the terrain of ever-shifting metaphor: slavery has afterlives (Hartman 2008, 6); it leaves a wake (Sharpe 2016). From this perspective, the somewhat mixed results found here are unexpected.

However, other research seems to be broadly more consistent with what is found here. One study finds that the direct economic consequences of enslavement had dissipated by 1940, whereas the consequences of Jim Crow—and thus also the indirect consequences of enslavement, by putting formerly enslaved families in what would become Jim Crow states—continue to this day (Althoff and Reichardt, n.d.). Another study suggests that the consequences of eradicating Jim Crow legal regimes for the survival of Black babies—a particularly consequential determinant of life expectancy—were immediate (Krieger et al. 2013).

To the extent that the results found here vary across measures, they raise a question about the extent to which parts of the legacy of slavery research program, in which studies typically rely on a single measure, may have a “file-drawer problem” (Rosenthal 1979). From the perspective of reparations programs, one implication of the results found here is that efforts to use such cross-state comparisons to calibrate metrics of lost lifespan probably need to commit to a measure on theoretical grounds, which may be difficult to justify.

### Interpreting Geographic Variation in Lifespans

The premise of relating Black lifespans to state-level Jim Crow, slavery, and racial terror is that the variation across states in the intensity of these regimes captures the way that those regimes structure contemporary lifespans. That premise may not be true. Indeed, both baselines that are implicated in these analyses—Black lifespans in other states and White lifespans in the same state—are problematic from the perspective of identifying how racial regimes and histories of enslavement and Jim Crow structure Black lifespans today. The problems with the northern baseline are discussed in the remainder of this section; the problems with the White baseline are elaborated elsewhere (Wrigley-Field, n.d.) but amount to the fact that White mortality is hardly unaffected by racism and the legacies of slavery (Metzl 2019; Malat, Mayorga-Gallo, and Williams 2018; McGhee 2022).^14^

The act of comparing Black lifespans across states based on states’ intensity of slavery and Jim Crow implicitly imagines that other states were fully outside that system. This is not true. To deny it is not to deny the uniqueness of the Jim Crow regime or to imply that living under that regime was no different from living in the North; Jim Crow was unique in its harms. Yet there is something odd about the premise that, on the one hand, slavery in the South still affects southern states today, but on the other hand, the fact that the entire country was founded on slavery no longer deeply affects the North. Indeed, Black Americans’ advances in freedom—whether emancipation (Downs 2012) or northward migration (Black et al. 2015)—often came at the cost of shortened lives, in the respective contexts of abandoned Reconstruction and the brutal segregation of northern cities (Leibbrand et al. 2020), among other racist policy responses in northern migration destinations (Derenoncourt 2022)—despite substantial Black organizing for health (Long 2016; Nelson 2011). It seems perverse, then, to use the very places where lifespans were shorter, where Black migrants bought freedom (from Jim Crow) with freedom (their lives), as a neutral measure of lifespan free of Jim Crow’s echoes today.

This problem highlights a distinctive challenge of conceptualizing redress for lost lifespan: the lack of a natural metric, which creates the need for a baseline against which to compare even if no good baseline exists. Attempts at enumerating the scale of slavery’s theft of wealth are plagued with challenges, but they are conceptually clearer. One approach that some authors have taken is to focus on a single pivotal moment and ask, how much wealth was stolen just through this one injustice alone? (Logan and Darity 2020) This strategy does not presume that no other atrocities matter or deserve redress; it simply makes the problem of beginning an accounting more tractable. It would be useful if comparing Black lifespans in northern and southern states provided a similarly revealing, if imperfect, starting point. But the studies of wealth stolen in (in that example) one race riot in Arkansas do not assume that the wealth distribution is otherwise unproblematic, whereas using Black lifespans in northern states—or in southern states that enslaved fewer people or passed fewer Jim Crow laws—as a baseline builds in the assumption that those lifespans were not limited by slavery and Jim Crow.

The analysis here explored a variety of measures of the intensity of slavery and Jim Crow to see whether they might offer clear targets for quantifying the degree of lost lifespan that should be redressed. In the end, the variable results across measures and the fact that these racial regimes, in addition to being particular to the South, were also integral to the United States as a whole, suggest that—though perhaps all American lifespans are shaped by slavery in some way—the most meaningful target may simply be the overall White-Black lifespan gap in the full United States.^15^ Before the COVID-19 pandemic, that gap was four years of life expectancy; during 2020 and 2021, it was nearly six years (Arias et al. 2022). In the remainder of this article, I consider what it could look like to take, as a target of redress, giving Black and White Americans equal access to time.

## WHAT AVENUES COULD REDRESS STOLEN TIME SPECIFICALLY?

If lifespan is the target of redress, what is the mechanism?

### Can Wealth-Based Reparations Amount to Health-Based Reparations?

In particular, can the mechanism be wealth-based reparations? We know two things clearly: one major pathway that produces ill health and shortened lifespans for Black people in the United States runs through inadequate income and wealth; and income and wealth are not the only source of the health and lifespan inequities that Black populations face.

That much lifespan inequity runs through wealth is clear; research has found that wealth, income, and other economic resources account for substantial portions of racial inequity in survival (Sudano and Baker 2006) and health (Hayward et al. 2000), though others find that such economic variables account for little racial disparity in certain important health outcomes, such as cardiovascular conditions (Teitler et al. 2021). Three studies stand out for results that suggest that wealth differences may drive nearly all of White-Black mortality disparities. The first (Geruso 2012) finds that the vast majority of the White-Black life expectancy gap, excluding infant mortality, can be accounted for statistically by economic and demographic variables, although the underlying samples are small relative to the task of estimating mortality in small age groups. The second (Himmelstein et al. 2022) finds that wealth differences can statistically account for mortality disparities above age fifty. The third (Do, Frank, and Finch 2012) finds that self-rated health can be fully accounted for by socioeconomic status when the latter is comprehensively accounted for. Other research, however, suggests that study designs that do not account for the race-specific consequences of socioeconomic position (and stressors) can overestimate those conditions’ contributions to racial health gaps, and that such contributions are substantial but far from total (Brown et al. 2023).

The findings of that third study, among others, may also suggest that wealth-based reparations might improve health in ways that research employing standard economic measures fails to capture. Limitations of conventional measures are (among others) that they measure the economic resources of individuals but not their community contexts (Do, Frank, and Finch 2012) and that they typically reflect a single moment in time (Boen 2016). In contrast, class evolves over the life course (Phillips, Martin, and Belmi 2020) and Black and White individuals with the same point-in-time economic status have likely held it for different lengths of time (Do, Frank, and Finch 2012; Boen 2016) and different numbers of generations (Sharkey 2008). An adequate wealth-based reparations program would provide flexible protection at all stages of life: a cushion against poverty, a way of limiting deleterious exposures, and the possibility of making investments across all of life, all benefiting recipients’ descendants as well as themselves.^16^

Finally, some research finds that racial disparities in death during key midlife ages—the ages when most of the population-level lost lifespan is concentrated (Bor et al. 2022)—occur exclusively among those with relatively low levels of flourishing (Louie et al. 2021), a psychosocial construct capturing holistic well-being (VanderWeele, McNeely, and Koh 2019; Levin 2021), and it is plausible that large-scale wealth redistribution would sharply curtail the prevalence of low flourishing, such as by limiting poverty (Desmond 2023; Linares, Kandasamy, and Vladutiu 2022) and enabling education (VanderWeele 2017). Thus, across many distinct lines of research, the idea that wealth engenders health seems nearly undeniable, and research offers substantial direct and indirect evidence that a radical redistribution of wealth toward Black Americans would provide similarly radical improvements in Black health.

Yet much research also finds substantial health and longevity gaps between Black and White Americans net of their economic position; indeed, some studies find that these gaps are largest among the wealthy (Colen 2011) and the most highly educated (Farmer and Ferraro 2005; Bell et al. 2020). Such findings have historical echoes: a study based on detailed collection of death certificates in the Carolinas across the twentieth century (Logan and Parman 2014) finds that, in the early twentieth century, Black workers in higher-status occupations had higher mortality than those in lower-status occupations—the inverse of the White pattern. Wealth does not protect against all forms of discrimination; indeed, it can intensify risk of certain kinds of discrimination by bringing Black people into settings in which few other people are Black (Colen et al. 2018; DeAngelis 2022) and where racial boundaries might be most actively defended (Christensen and Timmins 2023). These encounters are not benign; the evidence that experiences of discrimination can harm health through stress pathways is pervasive and convincing (Williams and Mohammed 2009; Lewis, Cogburn, and Williams 2015; Goosby, Cheadle, and Mitchell 2018), particularly for mental health outcomes (Paradies 2006), whose impacts on lifespan are indirect but likely meaningful, and whose impacts on the quality of life are clear.

Wealth-based reparations would not directly address one particular kind of discrimination with obvious import for health: discrimination by medical providers (Smedley, Stith, and Nelson 2003; Spencer and Grace 2016; Nong et al. 2020; McClure et al. 2020; Bavli and Jones 2022; Green et al. 2023; Brown et al. 2023), makers of medical devices (Kadambi 2021), and police and security guards in medical settings (Saadi and Ray 2023),^17^ leading Black patients to have worse diagnoses, treatments, and outcomes.^18^ Such mechanisms may explain the finding that Black Americans do not receive the same health returns on socioeconomic status that White Americans do (Boen 2016). Moreover, equivalent wealth will not necessarily provide the same health purchasing power to Black and White Americans; Black people typically pay more for goods and services of all kinds because of segregation (Williams, Priest, and Anderson 2016). Discrimination also limits the economic returns to wealth (Shapiro, Meschede, and Osoro 2013), particularly via smaller returns on housing investments (Thomas, Mann, and Meschede 2018).

To what extent do these lines of research bear on the question of what would happen if a major wealth-based reparations program were enacted? Research necessarily tells us in what ways wealth protects and fails to protect in the current context, in which racial wealth gaps are vast. It cannot directly tell us what wealth would or wouldn’t be able to buy in the vastly different context of a truly large infusion of wealth into a sizable portion of the U.S. Black population, an instance of the “SUTVA [stable unit treatment value assumption] problem” in causal inference (Morgan and Winship 2014, 48–55). For example, if wealth currently fails to protect because some of its benefits are offset by creating greater exposure to certain kinds of discrimination, it might protect more fully in a context in which wealthy spaces were more often Black spaces. More broadly, wealth redistribution could indirectly improve health by redistributing political power and might disrupt health consequences associated with being at the bottom of a social hierarchy, and positive feedback loops between increased health and improved political participation and democracy could result (Lynch 2023; Rodriguez 2018).

Consider neighborhoods. Neighborhoods are powerfully structured by income and wealth (Florida and Mellander 2015) and also powerfully structured by race independent of income and wealth (Taylor 2019; Coates 2014). Neighborhoods also seem to have some genuinely causal consequences for health, some of which flow through, for example, differential exposures to polluted air (Wodtke et al. 2022).^19^ To what extent would wealth-based reparations disrupt Black Americans’ differential exposure to pollutants? The answer seems to turn on the extent to which wealth allowed Black residents of the most polluted neighborhoods to move to safer ones (Pollack et al. 2023)—moves that, in the current context, are directly limited by discriminatory housing practices (Christensen, Sarmiento-Barbieri, and Timmins 2022)—and the extent to which a greater concentration of wealth increased Black communities’ ability to fight for their current neighborhoods to become less polluted (Currie, Voorheis, and Walker 2023; LaVeist 1992; Williams and Collins 1995, 377–78). In terms of this example, one way to cast the challenge posed in this article is this: Would articulating the lifespan gap as an independent target of reparations policy suggest that wealth transfers be supplemented with reparative environmental policies? If so, the deep links between place and health suggest that seeking to equalize lifespans might finally allow people to realize the twin mobility rights long denied by America’s racial regimes, the right to move freely and the right to stay where one is and have it be safe.

Finally, conceptualizing lost lifespan as a target of redress might serve an additional, expressive function: the social goods that follow from framing an injustice as something that needs repair (Russell 2022; Walker 2013).^20^ The experience of the 9/11 Victim Compensation Fund, as recounted by Gillian Hadfield (2008), may be instructive. Many families filed for compensation much later than expected, and legal experts interpreted this as reflecting disorganization or an expectation of a greater monetary payoff through a lawsuit. Interviews with families, however, revealed that their reluctance in fact reflected a series of nonmonetary goals that they weren’t sure could be achieved by the very large settlements being offered to them: information obtained through depositions, accountability for their losses, and political reforms that would reduce the chance of similar tragedies. The analogy may not hold. In the 9/11 case, people who had experienced a loss of great public concern had to decide “whether to resist the public characterization of their legal interests as private and monetary” (Hadfield 2008, 648); yet because the White-Black wealth gap is already socially defined as private, wealth-based reparations would seemingly reframe it as public. Lifespan-based reparations, of course, might also reframe that widely misunderstood disparity (Deyrup and Graves 2022) as well. Nevertheless, the 9/11 example illustrates that one yardstick for evaluating programs of repair is how fully they redress—or even acknowledge—the fullest extent of human freedoms that America’s history of enslavement still tramples.

### What Other Avenues Might There Be to Redress Lost Lifespan?

Proposals and policies that aim to redress short lifespans can adopt multiple strategies. They can target short lifespans directly, aiming to lengthen them. They can also aim to increase time freedom while people are still alive, perhaps granting greater control over time in total. Proposals can also be universal (indirectly racially equitable) or targeted, and targeted proposals can also be outcome-oriented or process-oriented (participatory). Whether universalist policies can count—in the context of an articulated motivation grounded in naming a specific harm (Walker 2013; Táíwò 2022)—as a type of reparation, or whether they can instead function as a meaningful alternative to reparations (Reed 2016; Taylor and Reed 2019) is, to say the least, debatable (Klein and Fouksman 2022; Russell 2022); but it is, perhaps, notable that the quintessential universalist American health program, Medicare, grew out of the civil rights movement (Smith 2016). In the next section, I provide some examples of types of policies that might provide some redress for stolen time, noting more and less targeted versions of policies that adopt a similar strategy to recovering time that has been taken. These possibilities, discussed in turn, are typologized in table 2.

One possibility involves a New Deal–style expansion of the Black medical and public health workforces and community health infrastructure. A community health infrastructure (Chowkwanyun 2022) could include creating permanent bases of funding and other support for community vaccination initiatives founded during the COVID-19 pandemic to expand their work into other health arenas (Faherty et al. 2021; Bile et al. 2022), perhaps drawing on models of infrastructure built around community organizations that developed to help HIV/AIDS patients (Kayal 1993); it could also draw from examples of medical-community collaborations to address the maternal health crisis for Black birthing people (Hardeman et al. 2020). Measures to radically expand the Black medical workforce could perhaps constitute a specific form of redress for the 1910 Flexner Report, which led to the closure of all but two Black medical schools; one consequence is the estimated loss of between ten thousand and thirty thousand Black medical graduates between the schools’ closure and 2019 (Campbell et al. 2020) and an unknown number of practitioners of adjacent medical professions (McLemore 2022, 51). To succeed, such efforts would need to also address factors that drive would-be Black medical and health workers out of their chosen professions, such as the “overpolicing” of Black medical residents (Ellis et al. 2023).

Evidence suggests that expanding the Black doctor pool could be extremely consequential for improving Black health. Research finds county-level correlations between Black primary care physicians and Black life expectancy (Snyder et al. 2023) and finds that Black new-borns are twice as likely to survive the days surrounding birth if they have a Black physician (Greenwood et al. 2020). Particularly compelling is a unique experiment based on an Oakland clinic that randomly assigned doctors of different races to Black male patients. Analysis of doctors’ notes suggested that the Black doctors had higher-quality interactions with their Black patients than other doctors did, and understood their health struggles more holistically in context of their life circumstances. Most strikingly, the study found results about patients’ willingness to embrace preventative measures that, if scaled up to the population, suggested that having enough Black doctors could reduce men’s Black-White gap in cardiovascular mortality by 19 percent (Alsan, Garrick, and Graziani 2019). Notably, however, the context in which Black doctors make such a significant difference is one in which Black men suffer so many risk factors for cardiovascular disease; in a context that successfully addressed the nonmedical sources of this disproportionate disease burden, Black doctors would have fewer lives to save.

This type of policy would be relatively targeted; more universalist versions could be policies such as Medicare for All or other single-payer health system, expanded enforcement of pollution laws, or measures designed to limit gun deaths. Despite the targeted nature of an expansion of the Black medical workforce specifically, any such expansion would likely also produce a model for similar expansions directed at the rural medical workforce, which might considerably improve White health and longevity as well (Simpson 2020; Gujral and Basu 2019).

A different approach would be to try to increase time freedom among the living. Universalist possibilities abound: fair scheduling laws (Schneider and Harknett 2019) and other workplace protections; efforts to aggressively reduce the administrative burden required to receive social services (Herd and Moynihan 2019; Herd et al. 2023; Jackson 2021); targeted improvements to transit infrastructure or expansion of work-proximity housing for populations with particularly burdensome commutes (Roberto 2008; Preston and McLafferty 2016); and policies, like Sunday closing laws, that promote access to predictable and shared time (Rose 2016), to name a few. A policy that could be implemented in a targeted or a universalist form would be a paid sabbatical program (Day 2019). Higher education faculty sabbaticals are, among other things, a form of work time that nevertheless has salutary effects on well-being (Davidson et al. 2010); a reparative sabbatical could, instead, be a paid period free of any work or governmental obligations, with the right to return to work guaranteed (as with leave, from some employers, under the Family Medical Leave Act, for example), in which sabbatical-takers could pursue whatever they chose.

Programs and policies that reduced time burden might also have multiplier effects through improving health and thus ultimately lifespans, as suggested by, for example, Cynthia Colen and colleagues’ (2023) systematic framework identifying time use as a social exposure that patterns health inequities. Lack of time is a major driver of unhealthy behaviors (Strazdins et al. 2011; Venn and Strazdins 2017; Covert 2022; Jabs and Devine 2006) and of stress (Mullainathan and Shafir 2021), and consistent, long-term reductions in time scarcity might plausibly shape the subjective time valuations that also influence health behaviors (Daugherty and Brase 2010). Parents’ and kin’s greater control over time in key moments in children’s and other loved ones’ lives might also produce spillover benefits to health (Stearns 2015).

A final category is participatory. Redress might take the form of a dedicated reparations fund, distinct from individual payments, meant to target lost time in particular. A collaborative process among those deemed eligible for individual payment, whether African descendants of slaves or some broader constituency (Jones 2021), could allow recipients to decide collectively what would do the most to return their time to them. Such a process could be modeled on examples of participatory budgeting (Fung and Wright 2011) and draw on lessons learned in analyses of victim compensation funds, such as the importance of recipients feeling like active participants (Feinberg 2005, 274–75). No one can better determine what would return time to the people it is being stolen from than those people themselves. Moreover, participatory processes could, perhaps uniquely, remain open to identifying—and seeking to ameliorate—further, potentially overlooked, dimensions of harm arising from slavery and Jim Crow that can still be redressed, for survivors and descendants, today.^21^

On the other hand, there may be something unsatisfactory about seeking to redress a population’s lost time through a process that demands of that same population that they take part in a time-consuming process of political participation (on the time demands of participatory democracy, see Cohen and Rogers 2003). Moreover, even though the strength of a participatory process is precisely its open-endedness, that very characteristic can be hard to maintain in practice. “The democratic process begins by defining the democratic body” (Demsas 2023) and the geographic scale at which participatory processes take place would likely circumscribe what kinds of options they could realistically consider.

## CONCLUSION

In *How the Word Is Passed,* his book on how slavery is remembered and forgotten in the United States, Clint Smith (2021, 104) quotes a poem that Mark King published in the Angola penitentiary’s prison magazine, *The Angolite*:

A century of forced labor, blood and pain.

Lives wasted, buried in shame.

Slavemasters oversee their daily tasks

Hidden behind century-old sadistic masks.

The world has passed this deathly land by.

The inhabitants still ask why.

“Lives wasted, buried in shame.” The poem evokes the memoirist and theorist Hafizah Augustus Geter: “I began to name my shame what it really was: America testing how long its history could last” (2022, xxvii). This is the fundamental harm that cries out for redress. Slavery was predicated on stealing human beings’ labor—their time and effort and creativity. This theft cannot be reduced to the theft of the wealth that they produced, because the ways that enslavement wasted lives were not only misdirected outputs, but also misdirected inputs: turning human beings’ most human qualities into instruments for others’ wealth and freedom at the expense of their own. The historian Walter Johnson (2016) puts this point like this (in an essay that Smith also draws on):

[The] language of “dehumanization” is misleading because slavery depended upon the human capacities of enslaved people. It depended upon their reproduction. It depended upon their labor. And it depended upon their sentience. Enslaved people could be taught: their intelligence made them valuable. They could be manipulated: their desires could make them pliable. They could be terrorized: their fears could make them controllable. And they could be tortured: beaten, starved, raped, humiliated, degraded. It is these last that are conventionally understood to be the most “inhuman” of slaveholders’ actions and those that most “dehumanized” enslaved people. And yet these actions epitomize the failure of this set of terms to capture what was at stake in slaveholding violence: the extent to which slaveholders depended upon violated slaves to bear witness, to provide satisfaction, to provide a living, human register of slaveholders’ power.

Johnson’s point, as I read it, is that a plantation owner or householder who obtained a wealth-making machine would not get all the things from it that they got from enslaving human beings.^22^ This point is relevant, though less relevant than that someone who had all their wealth stolen from them would not lose anything like what was taken from people who were enslaved. From both perspectives, wealth is a by-product of the true theft: the theft of humanity. There is something deeply limiting about reducing the meaning of stealing human beings’ lives to stealing the wealth that they produced.

Despite this limitation, wealth has—legitimately—been the focus of efforts at repair because wealth confers expansive, flexible power in a way that almost nothing else does. Perhaps a further reason that efforts at redress have focused on wealth is that it is transferable and fungible. Wealth is passed over generations; this is one major mechanism by which the theft of wealth hundreds of years ago creates injustice anew today, and it also creates a common-sense target for redress. But time does not transfer; no redress is possible for lives already cut short. That stark impossibility has no solution, and yet it also should inform the urgency with which any attempt at repair is undertaken; in the Evanston, Illinois, housing reparations program, six eligible would-be recipients have already died on the waiting list (Newton and Nelsen 2024).

At the end of Brittany Allen’s (2022) *Redwood*, about slavery’s reverberation through generations, the play’s main protagonist Meg poses a question of her ancestors: “I guess I wonder if they ever dreamed about *us*.” Four ancestors respond. Napoleon, the son of an enslaved woman and an enslaver, freed in his father’s will, says, “Dear clan: I did, I dreamed of you. Of course I dared to hope my descendants would know more freedom than I did. And your Great-Great-Great-Great Grandpa Napoleon would smile now to see you moving your beautiful brown bodies with abandon, to see the resilience that lives on in your cells.” But that enslaved woman herself, Napoleon’s mother Alameda, has a very different perspective, that culminates this way:

Did I dream about you?

When my tendons were slashed, my babies taken away?

no, children, no

there was no time for dreaming

He raped me for years and years.

I was never not pregnant, I was never not terrified, and as you cannot imagine my pain, I

cannot bless your pleasure

if I had it my way, y’all wouldn’t exist. None of you. For there can be nothing good across a

line like this.

As Alameda insists, no freedom for her son and his children and their children could redress what was done to her. There can be no redress for the theft of lifespans in the past. Yet, by the same token, no future redress will be possible for the lives being cut short now, for the millions of Black Americans who will die younger than they would had they lived and died like White people do, and who cannot live and die like White people do because they live in a country that built itself through enslaving Black people. The time for redress was in the past; it also is now.

## Supplementary Material

Appendix

## Figures and Tables

**Table 1.** Difference Between Bivariate Fitted-Value Lifespan Outcome at Racial Subjugation Measure’s 75th vs. 25th Percentile Values

	Black Life Expectancy	White Life Expectancy	Gap

1860 percent enslaved	0.10	*−1.85*	*−1.95*
1860 percent enslaved, Washington, D.C., excluded	−0.19	−0.36	−0.17
Jim Crow Laws passed before 1950	0.20	*−0.89*	*−1.09*
Jim Crow Laws, Washington, D.C., and Louisiana excluded	0.32	0.26	−0.07
Jim Crow school quality (reverse coded)	0.05	*−2.07*	*−2.11*
Jim Crow school quality (reverse coded), Washington, D.C., omitted	−0.30	−0.37	−0.07
Historical Racial Regimes index score	−0.18	0.25	0.43

*Source:* Author’s tabulation.

*Note:* Lifespans are life expectancies at the state level from 2017 to 2019 or, for lynchings with Black victims, smoothed life expectancies at the county level from 2015 to 2019. The analysis of percent enslaved is limited to states where it exceeds 1 percent. Italicized values are from regressions that are not preferred because they are heavily driven by very high White life expectancy in Washington, D.C. Table A.1 expands this table with results for two additional measures.

**Table 2.** Typology of Example Avenues of Address

	Targeted	Universal

Directed at lifespan	mass expansion of Black medical workforce	Medicare for All, pollution enforcement, true public safety measures
Directed at other time loss	paid sabbatical	fair scheduling laws, reduced administrative burdens in public services
Open	Participatory budgeting-style program (targeted or universal)	

*Source:* Author’s tabulation.

## Footnotes

1. In that sense, it is a complement to prior analyses such as Daina Ramey Berry’s (2017) assessment of the economic value of enslaved peoples and how those values may have intersected and contrasted with their internal value of themselves, “soul values” (61–66), and one another.

2. A few proposals, however, advocate for a broader set of health-promoting policies to address the legacies of slavery and Jim Crow (Gaskin, Headen, and White-Means 2004) and racist policies generally (Russell 2022; McLemore 2022), and for reparative policies to address the role of medical institutions in racist practice (Soled et al. 2021). Another tradition, not focused on health or lifespans, articulates reparations explicitly as more expansive than wealth transfers alone (Kelley 2022, 114; Táíwò 2022).

3. Lukas Althoff and Hugo Reichardt (n.d.) find that the contemporary economic gap between Black families whose ancestors were enslaved in 1860 and lived under Jim Crow regimes afterward and Black families who were free in 1860 and lived in non-Jim Crow states afterward is nearly half the size of the corresponding Black-White gap.

4. Age-adjusted White mortality was higher (worse) than Black pre-pandemic mortality in 2021, but not in 2020 or 2022. White life expectancy in each year of the COVID-19 pandemic was higher (better) than Black life expectancy has ever been (Wrigley-Field, n.d.).

5. Downs argues that “Freedpeople’s political mobilization for better health outcomes led local, state, and, most of all, the federal government to consider the health of the emancipated population as part of their responsibility to rebuild the South. . . . The Medical Division illustrates that freed slaves were the first advocates of federal health care” (Downs 2012, 167).

6. Many alternative procedures for translating lifespan into wealth are also possible. For example, such translations are built into cost-benefit analyses, including the “value of a statistical life” (Viscusi and Aldy 2003), which in the United States is presently estimated at around $10 million but varies with age, peaking in the forties and broadly following the age pattern of consumption (Kniesner and Viscusi 2019, 11–12). Similar translations are implicated in many other routine contexts, such as in the monetary penalties enacted by juries in civil court judgments related to wrongful deaths and in individuals’ decisions about life insurance (Friedman 2021). Such estimations can support an alternative set of metrics for developing wealth-based reparations (see Darity et al. 2024, this issue).

7. The profound symbolic importance of honoring the lives of those that have died—and of being unable to do so under threat of violence—also led Black funeral directors to play a distinctive and important role in the struggle for Black civil rights (Smith 2010; Parker 2023).

8. The numbers reported here consider states with 1860 enslaved populations versus all other states, including those that were not states in 1860. Readers who are rightly skeptical of some exceptionally high estimated life expectancies for Black populations in states where they are very small—Vermont and North Dakota—can take comfort that these values have little influence on the median values reported here. For the appendix, see https://www.rsfjournal.org/content/10/2/88/tab-supplemental.

9. Restricted-access data are needed to calculate race-specific life expectancy measures even for geographic units as large as states because death counts at childhood ages are often suppressed in public data.

10. Unlike the other measures, which are defined to include all states in 1860 or all states that employed Jim Crow policies, the HRR is defined only for states in the South as defined by the U.S. Census—which notably excludes Missouri, a slaveholding state in 1860 in which Black lifespans are notably low today—and further excludes Oklahoma (not yet incorporated in 1860) and Washington, D.C. An alternative version of the HRR, also developed by Baker (2022), produces essentially identical results in the present study and is not reported.

11. Historical lynchings, as measured in a different database from the one used in the appendix analyses, were previously found to be associated with county-level life expectancy (Kihlström and Kirby 2021) and with mortality rates for groups except Black men (Probst, Glover, and Kirksey 2019).

12. Lynch mob violence was also sometimes averted through “extraordinary interventions” in contexts where demands of the political economy made such violence undesirable to, for example, manufacturing interests (Beck, Tolnay, and Bailey 2016).

13. Other research identifies life expectancy costs associated with other forms of historical institutionalized racism, such as redlining (Graetz and Esposito 2023).

14. However, in contrast to the work just cited, see research by Ryan Gabriel and colleagues (2021) finding a protective effect of racism and histories of White racist violence on contemporary White opioid mortality.

15. As in the wealth gap, too, an alternative strategy, though daunting in practice, would be to attempt to comprehensively tabulate lifespan consequences of systemic racism, including persistent discrimination in housing, racial violence, and segregated public accommodations and public education—all common throughout the United States in the mid-twentieth century, not only in the South.

16. An additional challenge in generalizing from relationships between socioeconomic statuses and health outcomes in the current context to counterfactual contexts is that causes of death are also differentially susceptible to deleterious exposures at different life stages (see, for example, Leon and Walt 2000, 88–124) and large-scale changes in socioeconomic positions would presumably cause complex changes in the population distribution of health conditions and causes of death, with many additional consequences and feedback effects.

17. Notably, Altaf Saadi and Victor Ray’s (2023) analysis of newspaper accounts detailing patients’ experiences of police and security personnel violence in medical facilities found that health-care workers, as well as security workers, were implicated in many of the incidents (such as by calling on law enforcement to deal with combative patients). In other cases, hospital personnel (nurses, janitors, or other workers) were themselves the victims of police violence at work.

18. Medical research also substantially under-enrolls populations of color on average (Oh et al. 2015; *Scientific* *American* 2018), although the riskiest clinical research tends to overenroll vulnerable populations, including African Americans (Washington 2006), and mandates to deliberately increase racial and ethnic diversity in clinical trials are controversial (Epstein 2007).

19. Air pollution exposure in early childhood also appears to have causal consequences for subsequent White-Black earnings gaps, which indirectly implies health and cognitive consequences of pollution (Colmer, Voorheis, and Williams 2023).

20. Many ethical and political traditions—more broadly than in proposals for programs of reparations for slavery and institutionalized racism in the United States—identify naming, acknowledging, and accounting for harm as the foundational step in repair (Walker 2013; Colvin 2006; Ruttenberg 2022; Clarren 2023).

21. For example, time loss is itself only one form of a broader, multidimensional well-being, transcending wealth, that has been denied to enslaved people and, disproportionately, to their descendants. Benjamin Schneider (2022) argues that another consequential component of well-being is job quality, encompassing not only pay (and stability of employment) but also control, risk (to health), intensity, and repetitiveness; although it is not relevant to Schneider’s historical study, one might also imagine job meaningfulness. One might envision participatory processes with a broad remit seeking to address many distinct dimensions of harm to wellbeing in creative ways. This suggestion is broadly consistent with the approach taken by Olúfémi O. Táíwò (2022), who argues for aggressive decarbonization efforts as a form of reparations for slavery and colonialism.

22. The slavery scholar Saidiya Hartman makes a similar point about “the category crisis of human flesh and sentient commodity” differently: “In the archive of slavery, I encountered a paradox: the recognition of the slave’s humanity and status as a subject extended and intensified servitude and dispossession, rather than conferring some small measure of rights and protection. The attributes of the human—will, consciousness, reason, agency, and responsibility—were the inroads of discipline, punishment, and mortification. This paradox foreshadowed the subject of freedom and the limits of personhood bound indissolubly to property” (Hartman 2022).

## References

[R1] Acharya Avidit, Blackwell Matthew, and Sen Maya. 2016. “The Political Legacy of American Slavery.” The Journal of Politics 78(3): 621–41. 10.1086/686631.

[R2] Allen Brittany K. 2022. Redwood. Play. As performed at the Jungle Theater (Minneapolis) on February 27, 2022. Script courtesy of WME Agency.

[R3] Alsan Marcella, Garrick Owen, and Graziani Grant. 2019. “Does Diversity Matter for Health? Experimental Evidence from Oakland.” American Economic Review 109(12): 4071–11.

[R4] Althoff Lukas, and Reichardt Hugo. n.d. “Jim Crow and Black Economic Progress After Slavery.” Accessed March 1, 2023. https://lukasalthoff.github.io/jmp/althoff_jmp.pdf.

[R5] America Richard F., ed. 1990. The Wealth of Races: The Present Value of Benefits from Past Injustices. Contributions in Afro-American and African Studies, no. 132. New York: Greenwood Press.

[R6] Arias Elizabeth, Betzaida Tejada-Vera Kenneth Kochanek, and Ahmad Farida. 2022. “Provisional Life Expectancy Estimates for 2021.” Washington, D.C.: National Center for Health Statistics.

[R7] Baker Regina S. 2022. “The Historical Racial Regime and Racial Inequality in Poverty in the American South.” American Journal of Sociology 127(6): 1721–81.

[R8] Bassett Mary T., and Galea Sandro. 2020. “Reparations as a Public Health Priority—A Strategy for Ending Black–White Health Disparities.” New England Journal of Medicine 383(22): 2101–103. 10.1056/NEJMp2026170. 33031653

[R9] Bavli Itai, and Jones David S.. 2022. “Race Correction and the X-Ray Machine—The Controversy over Increased Radiation Doses for Black Americans in 1968.” New England Journal of Medicine 387(10): 947–52. 36069880 10.1056/NEJMms2206281

[R10] Beck EM, Tolnay Stewart E., and Bailey Amy Kate. 2016. “Contested Terrain: The State Versus Threatened Lynch Mob Violence.” American Journal of Sociology 121(6): 1856–84.

[R11] Bell Caryn N., Sacks Tina K., Thomas Tobin Courtney S., and Thorpe Roland J.. 2020. “Racial Non-Equivalence of Socioeconomic Status and Self-Rated Health Among African Americans and Whites.” SSM—Population Health 10 (April): 100561. 32140544 10.1016/j.ssmph.2020.100561 PMC7049651

[R12] Berry Daina Ramey. 2017. The Price for Their Pound of Flesh: The Value of the Enslaved from Womb to Grave in the Building of a Nation. Boston, Mass.: Beacon Press.

[R13] Bertocchi Graziella, and Dimico Arcangelo. 2012. “The Racial Gap in Education and the Legacy of Slavery.” Journal of Comparative Economics 40(4): 581–95.

[R14] Bile Ramla, Gilbert Ann, Mohamed Saida, Mohammed Inari, Plummer Matthew, and Wrigley-Field Elizabeth. 2022. “Lessons from an Immigrant-Focused Community COVID-19 Vaccination Organization.” Health Affairs Forefront, May. Accessed January 15, 2024. https://www.healthaffairs.org/content/forefront/lessons-immigrant-focused-community-covid-19-vaccination-organization.

[R15] Bilmes Linda, and Brooks Cornell William. 2024. “Normalizing Reparations: U.S. Precedent, Norms, and Models for Compensating Harms and Implications for Reparations to Black Americans.” RSF: The Russell Sage Foundation Journal of the Social Sciences 10(2): 30–68. 10.7758/RSF.2024.10.2.02.

[R16] Black Dan A., Sanders Seth G., Taylor Evan J., and Taylor Lowell J.. 2015. “The Impact of the Great Migration on Mortality of African Americans: Evidence from the Deep South.” American Economic Review 105(2): 477–503. 10.1257/aer.20120642. 26345146 PMC4559284

[R17] Boen Courtney. 2016. “The Role of Socioeconomic Factors in Black-White Health Inequities Across the Life Course: Point-in-Time Measures, Long-Term Exposures, and Differential Health Returns.” Social Science & Medicine 170 (December): 63–76. 27764654 10.1016/j.socscimed.2016.10.008 PMC5381512

[R18] Bor Jacob, Stokes Andrew C., Raifman Julia, Venkataramani Atheendar, Bassett Mary T., Himmelstein David, and Woolhandler Steffie. 2022. “Missing Americans: Early Death in the United States, 1933–2021.” Preprint. https://www.medrxiv.org/content/10.1101/2022.06.29.22277065v2.full.pdf. 10.1093/pnasnexus/pgad173 PMC10257439 37303714

[R19] Brown Crystal E., Marshall Arisa R., Snyder Cyndy R., Cueva Kristine L., Pytel Christina C., Jackson Sandra Y., Golden Sherita H., 2023. “Perspectives About Racism and Patient-Clinician Communication Among Black Adults with Serious Illness.” JAMA Network Open 6(7): e2321746. 10.1001/jamanetworkopen.2023.21746. 37405773 PMC10323709

[R20] Brown Tyson H., Hargrove Taylor W., Homan Patricia, and Adkins Daniel E.. 2023. “Racialized Health Inequities: Quantifying Socioeconomic and Stress Pathways Using Moderated Mediation.” Demography 60(3): 675–705. 37218993 10.1215/00703370-10740718 PMC10841571

[R21] Bruch Sarah K., Rosenthal Aaron J., and Soss Joe. 2019. “Unequal Positions: A Relational Approach to Racial Inequality Trends in the US States, 1940–2010.” Social Science History 43(1): 159–84. 10.1017/ssh.2018.36.

[R22] Burnham Margaret. 2022. By Hands Now Known: Jim Crow’s Legal Executioners. First edition. New York: W. W. Norton.

[R23] Campbell Kendall M., Corral Irma, Infante Linares Jhojana L., and Tumin Dmitry. 2020. “Projected Estimates of African American Medical Graduates of Closed Historically Black Medical Schools.” JAMA Network Open 3(8): e2015220. 32816033 10.1001/jamanetworkopen.2020.15220 PMC7441360

[R24] Card David, and Krueger Alan B.. 1992. “School Quality and Black-White Relative Earnings: A Direct Assessment.” Quarterly Journal of Economics 107(1): 151–200.

[R25] Chowkwanyun Merlin. 2022. All Health Politics Is Local: Community Battles for Medical Care and Environmental Health. Chapel Hill: University of North Carolina Press.

[R26] Christensen Peter, Sarmiento-Barbieri Ignacio, and Timmins Christopher. 2022. “Housing Discrimination and the Toxics Exposure Gap in the United States: Evidence from the Rental Market.” Review of Economics and Statistics 104(4): 807–18.

[R27] Christensen Peter, and Timmins Christopher. 2023. “The Damages and Distortions from Discrimination in the Rental Housing Market.” Quarterly Journal of Economics 138(4): 2505–57.

[R28] Rights Civil & Restorative Justice Project. n.d. “The Burnham-Nobles Digital Archive.” Accessed October 1, 2022. https://www.crrjarchive.org/crrj.

[R29] Clarren Rebecca. 2023. The Cost of Free Land: Jews, Lakota, and an American Inheritance. New York: Viking.

[R30] Coates Ta-Nehisi. 2014. “The Case for Reparations.” The Atlantic, June 2014. https://www.theatlantic.com/magazine/archive/2014/06/the-case-for-reparations/361631.

[R31] Cohen Elizabeth F. 2018. The Political Value of Time: Citizenship, Duration and Democratic Justice. Cambridge: Cambridge University Press.

[R32] ———. n.d. “Temporal Autonomy.” Unpublished manuscript.

[R33] Cohen Joshua, and Rogers Joel. 2003. “Power and Reason.” In Deepening Democracy: Institutional Innovations in Empowered Participatory Governance, edited by Fung Archon and Wright Erik Olin, 237–55. London: Verso.

[R34] Colen Cynthia G. 2011. “Addressing Racial Disparities in Health Using Life Course Perspectives: Toward a Constructive Criticism.” Du Bois Review: Social Science Research on Race 8(1): 79–94. 10.1017/S1742058X11000075.

[R35] Colen Cynthia G., Drotning Kelsey J., Sayer Liana C., and Link Bruce. 2023. “A Matter of Time: Racialized Time and the Production of Health Disparities.” Journal of Health and Social Behavior, June. Epub ahead of print. 10.1177/00221465231182377. PMC11912507 37377057

[R36] Colen Cynthia G., Ramey David M., Cooksey Elizabeth C., and Williams David R.. 2018. “Racial Disparities in Health among Nonpoor African Americans and Hispanics: The Role of Acute and Chronic Discrimination.” Social Science & Medicine 199 (February): 167–80. 28571900 10.1016/j.socscimed.2017.04.051 PMC5673593

[R37] Colmer Jonathan, Voorheis John, and Williams Brennan. 2023. “Air Pollution and Economic Opportunity in the United States.” Unpublished manuscript.

[R38] Colvin Christopher J. 2006. “Overview of the Reparations Program in South Africa.” In The Handbook of Reparations, edited by Greiff Pablo De, 176–215. Oxford University Press.

[R39] Covert Bryce. 2022. “The New Covid Boosters Are Incredible, and Everyone Should Get One.” New York Times, November 3. Accessed January 15, 2024. https://www.nytimes.com/2022/11/03/opinion/covid-booster-vaccine-poverty.html.

[R40] Craemer Thomas. 2015. “Estimating Slavery Reparations: Present Value Comparisons of Historical Multigenerational Reparations Policies: Estimating Slavery Reparations.” Social Science Quarterly 96(2): 639–55. 10.1111/ssqu.12151.

[R41] Cunningham David, Lee Hedwig, and Ward Geoff. 2021. “Legacies of Racial Violence: Clarifying and Addressing the Presence of the Past.” Annals of the American Academy of Political and Social Science 694(1): 8–20.

[R42] Currie Janet, Voorheis John, and Walker Reed. 2023. “What Caused Racial Disparities in Particulate Exposure to Fall? New Evidence from the Clean Air Act and Satellite-Based Measures of Air Quality.” American Economic Review 113(1): 71–97. 10.1257/aer.20191957.

[R43] Darity William Jr., Craemer Thomas, Berry Daina Ramey, and Francis Dania V.. 2024. “Black Reparations in the United States, 2024: An Introduction.” RSF: The Russell Sage Foundation Journal of the Social Sciences 10(2): 1–28. 10.7758/RSF.2024.10.2.01.

[R44] Darity William Jr., and Frank Dania. 2003. “The Economics of Reparations.” American Economic Review 93(2): 326–29. 10.1257/000282803321947281.

[R45] Darity William A. Jr., and Kirsten Mullen A. 2020. From Here to Equality: Reparations for Black Americans in the Twenty-First Century. Chapel Hill: University of North Carolina Press.

[R46] Darity William A. Jr., Mullen Kirsten, and Slaughter Marvin. 2022. “The Cumulative Costs of Racism and the Bill for Black Reparations.” Journal of Economic Perspectives 36(2): 99–122. 10.1257/jep.36.2.99.

[R47] Daugherty James R., and Brase Gary L.. 2010. “Taking Time to Be Healthy: Predicting Health Behaviors with Delay Discounting and Time Perspective.” Personality and Individual Differences 48(2): 202–207. 10.1016/j.paid.2009.10.007.

[R48] Davidson Oranit B., Eden Dov, Westman Mina, Yochi Cohen-Charash Leslie B. Hammer, Kluger Avraham N., Krausz Moshe, 2010. “Sabbatical Leave: Who Gains and How Much?” Journal of Applied Psychology 95(5): 953–64. 20718526 10.1037/a0020068

[R49] Day Meagan. 2019. “One Year Off, Every Seven Years.” Jacobin, May 22. Accessed January 15, 2024. https://jacobin.com/2019/05/workers-sabbatical-demand-leisure.

[R50] DeAngelis Reed T. 2022. “Moving on Up? Neighborhood Status and Racism-Related Distress Among Black Americans.” Social Forces 100(4): 1503–32. 10.1093/sf/soab075 PMC9285664 35847476

[R51] Demsas Jerusalem. 2023. “Local Government Has Too Much Power.” The Atlantic, July.

[R52] Derenoncourt Ellora. 2022. “Can You Move to Opportunity? Evidence from the Great Migration.” American Economic Review 112(2): 369–408.

[R53] Desmond Matthew. 2023. Poverty, by America. New York: Crown.

[R54] Deyrup Andrea, and Graves Joseph L.. 2022. “Racial Biology and Medical Misconceptions.” New England Journal of Medicine 386(6): 501–503. 35119803 10.1056/NEJMp2116224

[R55] Do D. Phuong, Frank Reanne, and Finch Brian Karl. 2012. “Does SES Explain More of the Black/White Health Gap than We Thought? Revisiting Our Approach Toward Understanding Racial Disparities in Health.” Social Science & Medicine 74(9): 1385–93. 22405688 10.1016/j.socscimed.2011.12.048

[R56] Doka Kenneth J. 1999. “Disenfranchised Grief.” Bereavement Care 18(3): 37–39.

[R57] Downs Jim. 2012. Sick from Freedom: African-American Illness and Suffering during the Civil War and Reconstruction. New York: Oxford University Press.

[R58] Duxbury Scott W. 2023. “Peculiar Institution? The Legacy of Slavery and Prison Expansion in the United States, 1970–2015.” Justice Quarterly. 10.1080/07418825.2023.2188073.

[R59] Dwyer-Lindgren Laura, Kendrick Parkes, Kelly Yekaterina O, Sylte Dillon O, Schmidt Chris, Blacker Brigette F, Daoud Farah, 2022. “Life Expectancy by County, Race, and Ethnicity in the USA, 2000–19: A Systematic Analysis of Health Disparities.” The Lancet 400(10345): 25–38. 10.1016/S0140-6736(22)00876-5. PMC9256789 35717994

[R60] Ellis Joshua, Otugo Onyeka, Landry Adaira, and Landry Alden. 2023. “Dismantling the Overpolicing of Black Residents.” New England Journal of Medicine 389(14): 1258–61. 37782019 10.1056/NEJMp2304559

[R61] Epstein Steven. 2007. Inclusion: The Politics of Difference in Medical Research. Chicago Studies in Practices of Meaning. Chicago: University of Chicago Press.

[R62] Faherty Laura J., Ringel Jeanne S., Williams Malcolm V., Kranz Ashley M., Perez Lilian, Schulson Lucy B., Gittens Allyson D., Phillips Brian, Baker Lawrence, Gandhi Priya, 2021. “Early Insights from the Equity-First Vaccination Initiative.” Santa Monica, Calif.: RAND Corporation. https://www.rand.org/pubs/research_briefs/RBA1627-1.html. 10.7249/rhq-10-02-03 PMC10187558 37200826

[R63] Farmer Melissa M., and Ferraro Kenneth F.. 2005. “Are Racial Disparities in Health Conditional on Socioeconomic Status?” Social Science & Medicine 60(1): 191–204. 15482878 10.1016/j.socscimed.2004.04.026

[R64] Feinberg Kenneth. 2005. “The Building Blocks of Successful Victim Compensation Programs.” Ohio State Journal on Dispute Resolution 20(1): 273–78.

[R65] Florida Richard, and Mellander Charlotta. 2015. “Segregated City: The Geography of Economic Segregation in America’s Metros.” Martin Prosperity Institute. https://www.diva-portal.org/smash/get/diva2:868382/FULLTEXT01.pdf.

[R66] Franke Katherine. 2019. Repair: Redeeming the Promise of Abolition. Chicago: Haymarket Books.

[R67] Friedman Howard Steven. 2021. Ultimate Price: The Value We Place on Life. Berkeley: University of California Press.

[R68] Fung Archon, and Wright Erik Olin. 2011. Deepening Democracy: Institutional Innovations in Empowered Participatory Governance. The Real Utopias Project 4. London: Verso.

[R69] Gabriel Ryan, Esposito Michael, Ward Geoff, Lee Hedwig, Hicken Margaret T., and Cunningham David. 2021. “White Health Benefits of Histories of Enslavement: The Case of Opioid Deaths.” Annals of the American Academy of Political and Social Science 694(1): 142–56. 10.1177/00027162211009776. PMC8386285 34446942

[R70] Gaskin Darrell J., Headen Alvin E., and White-Means Shelley I.. 2004. “Racial Disparities in Health and Wealth: The Effects of Slavery and Past Discrimination.” Review of Black Political Economy 32(3–4): 95–110. 10.1007/s12114-005-1007-9.

[R71] Geruso Michael. 2012. “Black-White Disparities in Life Expectancy: How Much Can the Standard SES Variables Explain?” Demography 49(2): 553–74. 10.1007/s13524-011-0089-1. 22287272

[R72] Geter Hafizah. 2022. The Black Period: On Personhood, Race, and Origin. New York: Random House.

[R73] Goodin Robert E., ed. 2008. Discretionary Time: A New Measure of Freedom. New York: Cambridge University Press.

[R74] Goosby Bridget J., Cheadle Jacob E., and Mitchell Colter. 2018. “Stress-Related Biosocial Mechanisms of Discrimination and African American Health Inequities.” Annual Review of Sociology 44(1): 319–40. 10.1146/annurev-soc-060116-053403. PMC10704394 38078066

[R75] Gottlieb Aaron, and Flynn Kalen. 2021. “The Legacy of Slavery and Mass Incarceration: Evidence from Felony Case Outcomes.” Social Service Review 95(1): 3–35.

[R76] Graetz Nick, and Esposito Michael. 2023. “Historical Redlining and Contemporary Racial Disparities in Neighborhood Life Expectancy.” Social Forces 102(1): 1–22.

[R77] Green Tiffany L., Vu Hoa, Swan Laura E.T., Luo Dian, Hickman Ellen, Plaisime Marie, and Hagiwara Nao. 2023. “Implicit and Explicit Racial Prejudice among Medical Professionals: Updated Estimates from a Population-Based Study.” The Lancet Regional Health—Americas 21 (May): 100489. 10.1016/j.lana.2023.100489. 37179794 PMC10172896

[R78] Greenwood Brad N., Hardeman Rachel R., Huang Laura, and Sojourner Aaron. 2020. “Physician-Patient Racial Concordance and Disparities in Birthing Mortality for Newborns.” Proceedings of the National Academy of Sciences 117(35): 21194–200. 10.1073/pnas.1913405117 PMC7474610 32817561

[R79] Gujral Kritee, and Basu Anirban. 2019. “Impact of Rural and Urban Hospital Closures on Inpatient Mortality.” w26182. Cambridge, MA: National Bureau of Economic Research.

[R80] Hadfield Gillian K. 2008. “Framing the Choice Between Cash and the Courthouse: Experiences with the 9/11 Victim Compensation Fund.” Law & Society Review 42(3): 645–82.

[R81] Hardeman Rachel R., Karbeah J Mag, Almanza Jennifer, and Kozhimannil Katy B.. 2020. “Roots Community Birth Center: A Culturally-C entered Care Model for Improving Value and Equity in Childbirth.” Healthcare 8(1): 100367. 10.1016/j.hjdsi.2019.100367. 31371235

[R82] Hartman Saidiya. 2008. Lose Your Mother: A Journey Along the Atlantic Slave Route. New York: Farrar, Straus & Giroux.

[R83] ———. 2022. “The Hold of Slavery.” New York Review of Books, October 24. https://www.nybooks.com/online/2022/10/24/the-hold-of-slavery-hartman.

[R84] Hayward Mark D., Miles Toni P., Crimmins Eileen M., and Yang Yu. 2000. “The Significance of Socioeconomic Status in Explaining the Racial Gap in Chronic Health Conditions.” American Sociological Review 65(6): 910. 10.2307/2657519.

[R85] Herd Pamela, Hoynes Hilary, Michener Jamila, and Moynihan Donald. 2023. “Introduction: Administrative Burden as a Mechanism of Inequality in Policy Implementation.” RSF: The Russell Sage Foundation Journal of the Social Sciences 9(4): 1–30. 10.7758/RSF.2023.9.4.01.

[R86] Herd Pamela, and Moynihan Donald P.. 2019. Administrative Burden: Policymaking by Other Means. New York: Russell Sage Foundation.

[R87] Himmelstein Kathryn E. W., Lawrence Jourdyn A., Jahn Jaquelyn L., Ceasar Joniqua N., Morse Michelle, Bassett Mary T., Wispelwey Bram P., Darity William A., and Venkataramani Atheendar S.. 2022. “Association Between Racial Wealth Inequities and Racial Disparities in Longevity Among US Adults and Role of Reparations Payments, 1992 to 2018.” JAMA Network Open 5(11): e224 0519. 10.1001/jamanetworkopen.2022.40519 PMC9641537 36342718

[R88] Jabs Jennifer, and Devine Carol M.. 2006. “Time Scarcity and Food Choices: An Overview.” Appetite 47(2): 196–204. 10.1016/j.appet.2006.02.014. 16698116

[R89] Jackson Michelle Victoria. 2021. Manifesto for a Dream: Inequality, Constraint, and Radical Reform. Stanford, Calif.: Stanford University Press.

[R90] Johnson Walter. 2016. “To Remake the World: Slavery, Racial Capitalism, and Justice.” Boston Review, October 26.

[R91] Jones William P. 2021. “What Is Owed.” The Nation, September 8.

[R92] Kadambi Achuta. 2021. “Achieving Fairness in Medical Devices.” Science 372(6537): 30–31. 33795446 10.1126/science.abe9195

[R93] Kayal Philip M. 1993. Bearing Witness: Gay Men’s Health Crisis and the Politics of AIDS. Boulder, Colo.: Westview Press.

[R94] Keele Luke, Cubbison William, and White Ismail. 2021. “Suppressing Black Votes: A Historical Case Study of Voting Restrictions in Louisiana.” American Political Science Review 115(2): 694–700. 10.1017/S0003055421000034.

[R95] Kelley Robin D. G. 2022. Freedom Dreams: The Black Radical Imagination, rev. ed. Boston, Mass.: Beacon Press.

[R96] Kihlström Laura, and Kirby Russell S.. 2021. “We Carry History Within Us: Anti-Black Racism and the Legacy of Lynchings on Life Expectancy in the U.S. South.” Health & Place 70 (July): 102618. 10.1016/j.healthplace.2021.102618. 34252751

[R97] Klein E, and Fouksman E. 2022. “Reparations as a Rightful Share: From Universalism to Redress in Distributive Justice.” Development and Change 53(1): 31–57.

[R98] Kniesner Thomas J., and Kip Viscusi W. 2019. “The Value of a Statistical Life.” In Oxford Research Encyclopedia of Economics and Finance, by Kniesner Thomas J. and Kip Viscusi W. Oxford: Oxford University Press. 10.1093/acrefore/9780190625979.013.138.

[R99] Konzcal Mike. 2021. “Time Is the Universal Measure of Freedom.” Boston Review, January 11. https://bostonreview.net/articles/mike-konczal-tk/.

[R100] Krieger Nancy, Chen Jarvis T., Coull Brent, Waterman Pamela D., and Beckfield Jason. 2013. “The Unique Impact of Abolition of Jim Crow Laws on Reducing Inequities in Infant Death Rates and Implications for Choice of Comparison Groups in Analyzing Societal Determinants of Health.” American Journal of Public Health 103(12): 2234–44. 24134378 10.2105/AJPH.2013.301350 PMC3828968

[R101] LaVeist Thomas A. 1992. “The Political Empowerment and Health Status of African-Americans: Mapping a New Territory.” American Journal of Sociology 97(4): 1080–95.

[R102] Lawrenz Kendall. 2022. “Remedying the Health Implications of Structural Racism Through Reparations.” George Washington Law Review 90(4): 1018–51.

[R103] Leibbrand Christine, Massey Catherine, Trent Alexander J, Genadek Katie R., and Tolnay Stewart. 2020. “The Great Migration and Residential Segregation in American Cities During the Twentieth Century.” Social Science History 44(1): 19–55. 32546874 10.1017/ssh.2019.46 PMC7297198

[R104] Leon David, and Walt Gill. 2000. Poverty, Inequality, and Health. Oxford: Oxford University Press.

[R105] Levin Jeff. 2021. “Human Flourishing: A New Concept for Preventive Medicine.” American Journal of Preventive Medicine 61(5): 761–64. 34187711 10.1016/j.amepre.2021.04.018

[R106] Lewis Tené T., Cogburn Courtney D., and Williams David R.. 2015. “Self-Reported Experiences of Discrimination and Health: Scientific Advances, Ongoing Controversies, and Emerging Issues.” Annual Review of Clinical Psychology 11(1): 407–40. 10.1146/annurev-clinpsy-032814-112728 PMC5555118 25581238

[R107] Linares Deborah E., Kandasamy Veni, and Vladutiu Catherine J.. 2022. “Lifecourse Factors Associated with Flourishing among US Children Aged 1–5 Years.” Child: Care, Health and Development 48(2): 298–310. 10.1111/cch.12930. 34791734

[R108] Logan Trevon D., and Parman John M.. 2014. “The Dynamics of African-American Health: A Historical Perspective.” Review of Black Political Economy 41(3): 299–318.

[R109] Logan Trevon D., and Darity William A. Jr. 2020. “Look What Has Been Taken From Black Americans.” Bloomberg, September 21.

[R110] Long Margaret Geneva. 2016. Doctoring Freedom: The Politics of African American Medical Care in Slavery and Emancipation. Chapel Hill: University of North Carolina Press.

[R111] Louie Patricia, Upenieks Laura, Siddiqi Arjumand, Williams David R., and Takeuchi David T.. 2021. “Race, Flourishing, and All-Cause Mortality in the United States, 1995–2016.” American Journal of Epidemiology 190(9): 1735–43. 33728457 10.1093/aje/kwab067 PMC8579008

[R112] Lynch Julia. 2023. “The Political Economy of Health: Bringing Political Science In.” Annual Review of Political Science 26(1): 389–410. 10.1146/annurev-polisci-051120-103015.

[R113] Malat Jennifer, Mayorga-Gallo Sarah, and Williams David R.. 2018. “The Effects of Whiteness on the Health of Whites in the USA.” Social Science & Medicine 199 (February): 148–56. 28716453 10.1016/j.socscimed.2017.06.034

[R114] Marx Karl. (1867) 1981. Capital: A Critique of Political Economy, edited by Fowkes Ben and Fernbach David. First published 1867 in Berlin. New York: Penguin Books in association with New Left Review.

[R115] McClure Elizabeth S, Vasudevan Pavithra, Bailey Zinzi, Patel Snehal, and Robinson Whitney R.. 2020. “Racial Capitalism Within Public Health—How Occupational Settings Drive COVID-19 Disparities.” American Journal of Epidemiology 189(11): 1244–53. 32619007 10.1093/aje/kwaa126 PMC7337680

[R116] McGhee Heather C. 2022. The Sum of Us: What Racism Costs Everyone and How We Can Prosper Together. New York: One World.

[R117] McLemore Monica R. 2022. “A Bolder Approach to Diversity the Health Care Workforce.” In The Black Agenda: Bold Solutions for a Broken System, edited by Opoku-Agyeman Anna Gifty, 47–55. New York: St. Martin’s Griffin.

[R118] Metzl Jonathan. 2019. Dying of Whiteness: How the Politics of Racial Resentment Is Killing America’s Heartland. New York: Basic Books.

[R119] Morgan Stephen L., and Winship Christopher. 2014. Counterfactuals and Causal Inference: Methods and Principles for Social Research, 2nd ed. Cambridge University Press.

[R120] Mullainathan Sendhil, and Shafir Eldar. 2021. Scarcity: The New Science of Having Less and How It Defines Our Lives. New York: Henry Holt.

[R121] Muller Christopher. 2021. “Exclusion and Exploitation: The Incarceration of Black Americans from Slavery to the Present.” Science 374(6565): 282–86. 34648320 10.1126/science.abj7781

[R122] Murray Pauli. (1950) 2016. States’ Laws on Race and Color. Athens: University of Georgia Press.

[R123] Nelson Alondra. 2011. Body and Soul: The Black Panther Party and the Fight Against Medical Discrimination. Minneapolis: University of Minnesota Press.

[R124] Newton Monique, and Nelsen Matthew D.. 2024. “The Politics of Expedience: Evanston, Illinois, and the Fight for Reparations.” RSF: The Russell Sage Foundation Journal of the Social Sciences 10(3): 114–39. 10.7758//RSF.2024.10.3.06.

[R125] Nong Paige, Raj Minakshi, Creary Melissa, Kardia Sharon L. R., and Platt Jodyn E.. 2020. “Patient-Reported Experiences of Discrimination in the US Health Care System.” JAMA Network Open 3(12): e2029650. 10.1001/jamanetworkopen.2020.29650. 33320264 PMC7739133

[R126] Oakes James. 2015. “Slavery Is Theft.” Jacobin, August 13. Accessed January 15, 2024. https://jacobin.com/2015/08/slavery-abolition-lincoln-oakes-property.

[R127] O’Connell Heather A. 2012. “The Impact of Slavery on Racial Inequality in Poverty in the Contemporary U.S. South.” Social Forces 90(3): 713–34. 10.1093/sf/sor021.

[R128] O’Connell Heather A., Curtis Katherine J., and DeWaard Jack. 2020. “Population Change and the Legacy of Slavery.” Social Science Research 87 (March): 102413. 32279864 10.1016/j.ssresearch.2020.102413 PMC13116197

[R129] Oh Sam S., Galanter Joshua, Thakur Neeta, Maria Pino-Yanes Nicolas E. Barcelo, White Marquitta J., De Bruin Danielle M., 2015. “Diversity in Clinical and Biomedical Research: A Promise Yet to Be Fulfilled.” PLOS Medicine 12(12): e1001918. 26671224 10.1371/journal.pmed.1001918 PMC4679830

[R130] Outterson Kevin. 2005. “Tragedy & Remedy: Reparations for Disparities in Black Health.” DePaul Journal of Health Care Law 9(1): 735–91. 17061385

[R131] ———. 2008. “The End of Reparations Talk: Reparations in an Obama World.” University of Kansas Law Review 57(4): 935–48.

[R132] Paradies Yin. 2006. “A Systematic Review of Empirical Research on Self-Reported Racism and Health.” International Journal of Epidemiology 35(4): 888–901. 16585055 10.1093/ije/dyl056

[R133] Parker David B. 2023. “How Black Americans Combated Racism from beyond the Grave.” The Conversation, June 14. Accessed January 15, 2024. http://theconversation.com/how-black-americans-combated-racism-from-beyond-the-grave-207200.

[R134] Petersen Nick, and Ward Geoff. 2015. “The Transmission of Historical Racial Violence: Lynching, Civil Rights–Era Terror, and Contemporary Interracial Homicide.” Race and Justice 5(2): 114–43. 10.1177/2153368714567577.

[R135] Phillips L. Taylor, Martin Sean R., and Belmi Peter. 2020. “Social Class Transitions: Three Guiding Questions for Moving the Study of Class to a Dynamic Perspective.” Social and Personality Psychology Compass 14(9): 1–18. 10.1111/spc3.12560.

[R136] Pollack Craig Evan, Roberts Laken C., Peng Roger D., Cimbolic Pete, Judy David, Balcer-Whaley Susan, Grant Torie, 2023. “Association of a Housing Mobility Program With Childhood Asthma Symptoms and Exacerbations.” JAMA 329(19): 1671. 37191703 10.1001/jama.2023.6488 PMC10189571

[R137] Preston Valerie, and Sara McLafferty. 2016. “Revisiting Gender, Race, and Commuting in New York.” Annals of the American Association of Geographers 106(2): 300–10.

[R138] Probst Janice C., Glover Saundra, and Kirksey Victor. 2019. “Strange Harvest: A Cross-Sectional Ecological Analysis of the Association Between Historic Lynching Events and 2010–2014 County Mortality Rates.” Journal of Racial and Ethnic Health Disparities 6(1): 143–52. 10.1007/s40615-018-0509-7. 30047094

[R139] Reece Robert L. 2022. “Slave Past, Modern Lives: An Analysis of the Legacy of Slavery and Contemporary Life Expectancy in the American South.” Journal of Black Studies 53(7): 677–702. 10.1177/00219347221095167.

[R140] Reed Adolph Jr. 2016. “The Case Against Reparations.” Nonsite.org (blog). February 11, 2016. https://nonsite.org/the-case-against-reparations.

[R141] Richardson Eugene T., Malik Momin M., Darity William A., Kirsten Mullen A, Morse Michelle E., Malik Maya, Maybank Aletha, 2021. “Reparations for Black American Descendants of Persons Enslaved in the U.S. and Their Potential Impact on SARS-CoV-2 Transmission.” Social Science & Medicine 276 (May): 113741. 33640157 10.1016/j.socscimed.2021.113741 PMC7871902

[R142] Roberto Elizabeth. 2008. “Commuting to Opportunity: The Working Poor and Commuting in the United States.” Washington, D.C.: Brookings Institution. Accessed January 15, 2024. https://www.brookings.edu/articles/commuting-to-opportunity-the-working-poor-and-commuting-in-the-united-states.

[R143] Rodriguez Javier M. 2018. “Health Disparities, Politics, and the Maintenance of the Status Quo: A New Theory of Inequality.” Social Science & Medicine 200 (March): 36–43. 29421470 10.1016/j.socscimed.2018.01.010

[R144] Rodriques Elias. 2022. “How Saidiya Hartman Changed the Study of Black Life (Interview with Sadiya Hartman).” The Nation, November 3.

[R145] Rose Julie L. 2016. Free Time. Princeton, N.J.: Princeton University Press.

[R146] Rosenthal Robert. 1979. “The File Drawer Problem and Tolerance for Null Results.” Psychological Bulletin 86(3): 638–41. 10.1037/0033-2909.86.3.638.

[R147] Russell Camisha. 2022. “The Case for Health Care Reparations.” Paper presented at the Ethics Grand Rounds, University of Minnesota Center for Bioethics, Minneapolis (October 21, 2022).

[R148] Ruttenberg Danya. 2022. On Repentance and Repair: Making Amends in an Unapologetic World. Boston, Mass.: Beacon Press.

[R149] Saadi Altaf, and Ray Victor E.. 2023. “Police Violence in Health Care Settings in US Media Coverage.” JAMA Network Open 6(11): e2342998. 37955898 10.1001/jamanetworkopen.2023.42998 PMC10644214

[R150] Schneider Benjamin. 2022. “Good Jobs and Bad Jobs in History.” Oxford Economic and Social History working paper no. 202. Oxford: Oxford University Press.

[R151] Schneider Daniel, and Harknett Kristen. 2019. “Consequences of Routine Work-Schedule Instability for Worker Health and Well-Being.” American Sociological Review 84(1): 82–114. 33311716 10.1177/0003122418823184 PMC7730535

[R152] American Scientific. 2018. “Clinical Trials Have Far Too Little Racial and Ethnic Diversity.” Editorial, September 1. Accessed January 15. 2024. 10.1038/scientificamerican0918-10.

[R153] Seguin Charles, and Rigby David. 2019. “National Crimes: A New National Data Set of Lynchings in the United States, 1883 to 1941.” Socius: Sociological Research for a Dynamic World 5 (January): 1–9. 10.1177/237802311984178.

[R154] Shapiro Thomas, Meschede Tatjana, and Osoro Sam. 2013. “The Roots of the Widening Racial Wealth Gap: Explaining the Black-White Economic Divide.” IASP Research and Policy Brief, June. Waltham, Mass: Brandeis University, Institute on Assets and Social Policy. Accessed January 15, 2024. https://heller.brandeis.edu/iere/pdfs/racial-wealth-equity/racial-wealth-gap/roots-widening-racial-wealth-gap.pdf.

[R155] Sharkey Patrick. 2008. “The Intergenerational Transmission of Context.” American Journal of Sociology 113(4): 931–69. 10.1086/522804.

[R156] Sharpe Christina Elizabeth. 2016. In the Wake: On Blackness and Being. Durham, NC: Duke University Press.

[R157] Simpson Kathleen Rice. 2020. “Ongoing Crisis in Lack of Maternity Services in Rural America.” MCN: The American Journal of Maternal/Child Nursing 45(2): 132. 32097229 10.1097/NMC.0000000000000605

[R158] Smedley Brian D., Adrienne Y. Stith, and Alan R. Nelson, eds. 2003. Unequal Treatment: Confronting Racial and Ethnic Disparities in Health Care (with CD). Washington, D.C.: National Academies Press. 10.17226/12875. 25032386

[R159] Smith Clint. 2021. How the Word Is Passed: A Reckoning with the History of Slavery across America. New York: Little, Brown.

[R160] Smith David Barton. 2016. The Power to Heal: Civil Rights, Medicare, and the Struggle to Transform America’s Health Care System. Nashville, Tenn.: Vanderbilt University Press.

[R161] Smith Suzanne E. 2010. To Serve the Living: Funeral Directors and the African American Way of Death. Cambridge, Mass.: Harvard University Press.

[R162] Snyder John E., Upton Rachel D., Hassett Thomas C., Lee Hyunjung, Nouri Zakia, and Dill Michael. 2023. “Black Representation in the Primary Care Physician Workforce and Its Association With Population Life Expectancy and Mortality Rates in the US.” JAMA Network Open 6(4): e236687. 10.1001/jamanetworkopen.2023.6687. 37058307 PMC10105312

[R163] Soled Derek Ross, Chatterjee Avik, Olveczky Daniele, and Lindo Edwin G.. 2021. “The Case for Health Reparations.” Frontiers in Public Health 9 (July): 664783. 34336763 10.3389/fpubh.2021.664783 PMC8323065

[R164] Spencer Karen Lutfey, and Grace Matthew. 2016. “Social Foundations of Health Care Inequality and Treatment Bias.” Annual Review of Sociology 42(1): 101–20.

[R165] Stearns Jenna. 2015. “The Effects of Paid Maternity Leave: Evidence from Temporary Disability Insurance.” Journal of Health Economics 43 (September): 85–102. 26218984 10.1016/j.jhealeco.2015.04.005

[R166] Strazdins Lyndall, Griffin Amy L., Broom Dorothy H., Banwell Cathy, Korda Rosemary, Dixon Jane, Paolucci Francesco, and Glover John. 2011. “Time Scarcity: Another Health Inequality?” Environment and Planning A: Economy and Space 43(3): 545–59.

[R167] Sudano Joseph J., and Baker David W.. 2006. “Explaining US Racial/Ethnic Disparities in Health Declines and Mortality in Late Middle Age: The Roles of Socioeconomic Status, Health Behaviors, and Health Insurance.” Social Science & Medicine 62(4): 909–22. 16055252 10.1016/j.socscimed.2005.06.041

[R168] Taifa Nkechi. 2020. “Let’s Talk About Reparations.” Columbia Journal of Race and Law 10 (1): Special Feature. 10.7916/CJRL.V10I1.5182.

[R169] Táíwò Olúfémi O. 2022. Reconsidering Reparations: Worldmaking in the Case of Climate Crisis. New York: Oxford University Press.

[R170] Taylor Keeanga-Yamahtta. 2019. Race for Profit: How Banks and the Real Estate Industry Undermined Black Homeownership. Chapel Hill: University of North Carolina Press.

[R171] Taylor Keeanga-Yamahtta, and Reed Adolph Jr. 2019. “The Reparations Debate.” Dissent, June 24. Accessed January 15, 2024. https://www.dissentmagazine.org/online_articles/the-reparations-debate.

[R172] Teitler Julien, Wood Bethany M., Zeng Weiwen, Martinson Melissa L., Plaza Rayven, and Reichman Nancy E.. 2021. “Racial-Ethnic Inequality in Cardiovascular Health in the United States: Does It Mirror Socioeconomic Inequality?” Annals of Epidemiology 62 (October): 84–91. 10.1016/j.annepidem.2021.04.019. 33991659 PMC8941185

[R173] Thakur Neeta, and Martinez Adali. 2023. “Housing Reparations as an Avenue to Counter the Impact of Structural Racism on Asthma.” JAMA 329(19): 1645. 37191711 10.1001/jama.2023.7242

[R174] Thomas Hannah, Mann Alexis, and Meschede Tatjana. 2018. “Race and Location: The Role Neighborhoods Play in Family Wealth and Well-Being.” American Journal of Economics and Sociology 77(3–4): 1077–111. 10.1111/ajes.12239.

[R175] Troesken Werner, and Walsh Randall. 2019. “Collective Action, White Flight, and the Origins of Racial Zoning Laws.” Journal of Law, Economics, and Organization 35(2): 289–318. 10.1093/jleo/ewz006.

[R176] University of Wisconsin Population Health Institute. n.d. “County Health Rankings and Roadmaps.” Accessed November 1, 2022. https://www.countyhealthrankings.org/explore-healthrankings/rankings-data-documentation/national-data-documentation-2010-2019.

[R177] VanderWeele Tyler J. 2017. “On the Promotion of Human Flourishing.” Proceedings of the National Academy of Sciences 114(31): 8148–56. 10.1073/pnas.1702996114. PMC5547610 28705870

[R178] VanderWeele Tyler J., McNeely Eileen, and Koh Howard K.. 2019. “Reimagining Health—Flourishing.” JAMA 321(17): 1667. 10.1001/jama.2019.3035. 30933213

[R179] Venn Danielle, and Strazdins Lyndall. 2017. “Your Money or Your Time? How Both Types of Scarcity Matter to Physical Activity and Healthy Eating.” Social Science & Medicine 172 (January): 98–106. 10.1016/j.socscimed.2016.10.023. 27839899

[R180] Viscusi W. Kip, and Aldy Joseph E.. 2003. “The Value of a Statistical Life: A Critical Review of Market Estimates Throughout the World.” Journal of Risk and Uncertainty 27(1): 5–76.

[R181] Walker Margaret Urban. 2013. “The Expressive Burden of Reparations: Putting Meaning into Money, Words, and Things.” In Justice, Responsibility and Reconciliation in the Wake of Conflict, edited by MacLachlan Alice and Speight Allen, 205–25. Dordrecht: Springer Netherlands. 10.1007/978-94-007-5201-6_12.

[R182] Ward Matthew. 2022. “The Legacy of Slavery and Contemporary Racial Disparities in Arrest Rates.” Sociology of Race and Ethnicity 8(4): 534–52.

[R183] Washington Harriet A. 2006. Medical Apartheid: The Dark History of Medical Experimentation on Black Americans from Colonial Times to the Present. New York: Harlem Moon.

[R184] Williams David R., and Collins Chiquita. 1995. “US Socioeconomic and Racial Differences in Health: Patterns and Explanations.” Annual Review of Sociology 21(1): 349–86.

[R185] Williams David R., and Collins Chiquita. 2004. “Reparations: A Viable Strategy to Address the Enigma of African American Health.” American Behavioral Scientist 47(7): 977–1000.

[R186] Williams David R., and Mohammed Selina A.. 2009. “Discrimination and Racial Disparities in Health: Evidence and Needed Research.” Journal of Behavioral Medicine 32(1): 20–47. 19030981 10.1007/s10865-008-9185-0 PMC2821669

[R187] Williams David R., Priest Naomi, and Anderson Norman B.. 2016. “Understanding Associations Among Race, Socioeconomic Status, and Health: Patterns and Prospects.” Health Psychology 35(4): 407–11. 10.1037/hea0000242. 27018733 PMC4817358

[R188] Williams Jhacova A., Logan Trevon D., and Hardy Bradley L.. 2021. “The Persistence of Historical Racial Violence and Political Suppression: Implications for Contemporary Regional Inequality.” Annals of the American Academy of Political and Social Science 694(1): 92–107. 10.1177/00027162211016298.

[R189] Wodtke Geoffrey T., Ard Kerry, Bullock Clair, White Kailey, and Priem Betsy. 2022. “Concentrated Poverty, Ambient Air Pollution, and Child Cognitive Development.” Science Advances 8(48): eadd0285. 10.1126/sciadv.add0285. 36449613 PMC9710878

[R190] Wrigley-Field Elizabeth. 2020. “US Racial Inequality May Be as Deadly as COVID-19.” Proceedings of the National Academy of Sciences 117(36): 21854–56. 10.1073/pnas.2014750117 PMC7486779 32839337

[R191] ———. n.d. “Mortality Differences as Racial Inequality.” Unpublished manuscript.

