# Supplementary material for: Stolen Lives: Redress for Slavery’s and Jim Crow’s Ongoing Theft of Lifespan
Source: RSF. Author manuscript; Available in PMC 2025 Jun 1. (PMC11801221; doi:10.7758/rsf.2024.10.2.04)
Supplement: Appendix [file NIHMS2048363-supplement-Appendix.pdf]

APPENDIX TO

Stolen Time: Redress for Slavery's and Jim Crow's Ongoing Theft of Lifespan

This Appendix presents more detailed empirical results about how Black life expectancy, white life expectancy, and the White–Black life expectancy gap vary with the intensity of slavery, Jim Crow, and racial terror across states and, in one case, counties. As context, Figure A1 illustrates visually the distribution of state-level lifespans by enslavement histories, as discussed in the text.

Additional measures

This Appendix repeats the empirical analysis using two additional measures, both designed to capture racial terror.

First, I examine counts of Jim Crow violent incidents, collected (from historical newspaper records) by the Burnham-Nobles Digital Archive (The Civil Rights & Restorative Justice Project, n.d.).[1] This dataset contains fewer states than the others analyzed here. Because the Burnham-Nobles data collection has proceeded state by state, it is important that missing data are not treated as implicitly zero (in contrast with, for example, the percent enslaved, where states that had already banned slavery are known, true zeroes).

Notably, the Burnham-Nobles measure of Jim Crow violent incidents is only loosely correlated with the state-level measures reported in the main text. While the Historic Racial Regimes index was moderately to highly correlated with the other measures, its correlation with the Burnham-Nobles measure is only 0.34. The Burnham-Nobles measure is the most correlated

[1] The Burnham-Nobles archive also contains a count of victims of Jim Crow violence; in the present study, this produces results essentially identical to those produced from the count of violent incidents, and is not reported.

with the 1860 proportion enslaved (.45) and the least correlated with the count of Jim Crow laws (-.07). The Burnham-Nobles measure is defined for the fewest states (11); the proportion enslaved is defined for the most (20 for which the proportion exceeded zero; 41 states and territories in total, reflecting the size and composition of the United States in 1860).

Second, I examine a single county-level measure: a count of lynchings of Black Americans in 1883-1941, as previously reconstructed from newspaper archives (Seguin and Rigby 2019). Prior analyses have found little meaningful state-level variation in lynchings, and have argued that the meaningful variation is at the broad regional and county levels (Seguin and Rigby 2019). The results presented below omit counties with zero reported Black lynchings, which may reflect either a lack of lynchings or a lack of newspaper coverage of the county during certain periods of time; results (below) show that including counties with zero lynchings generates qualitatively similar, but larger, relationships between the count of lynchings and the lifespan outcomes analyzed here.

Both of these measures rely on historical newspaper archives as their underlying data. Such archives produce distinctive challenges because White newspapers often did not cover racial terror, particularly before the interwar era. Meanwhile, Black newspapers produced courageous and careful documentation of lynchings and other racial violence long before liberal White southern editors and national editors followed suit (Roberts and Klibanoff 2007). But they did so with limited resources and in conditions of violent repression—"those who insisted on telling the whole truth paid a price" (Kelley 2021, 286–87)—that limited the geographic and topical scope of their coverage, as many brave editors and journalists fled north and those that remained in the South operated in an environment of censorship and control (including from the prominent Black leader Booker T. Washington (Bedingfield and Forde 2021, 8–9)). These

limitations of historical newspaper coverage raise particular worries for the current study's usage of the Burnham-Nobles archive, the strength of which is its inclusion of a wide array of acts of terror, not only those—lynchings and race riots—distinguished by mass participation.

Indeed, these limitations may apply less forcefully to documented lynchings than to more prosaic forms of violence and terror. Although white journalists did not always see lynchings as newsworthy in the 1880s, often mentioning them as ordinary events, this changed dramatically as the press reshaped itself in the context of radically expanded railroad networks throughout the South in the early twentieth century (Brundage 2021, 85–86). And while lynchings were, by the 1930s, "a routine, everyday sort of villainy" (W.T. Howard, quoted in Perloff (2000, 315)), nevertheless, "newspapers in every region of the country provided graphic coverage of lynchings, especially those that occurred in their area" (Perloff 2000, 319); in fact, newspapers competed to cover—in sensational style—lynchings and other "timely news from throughout their actual or aspirational circulation area. […] Indeed, an entire media cycle for lynching stories developed" (Brundage 2021, 86).

One additional basis for confidence in lynching counts is the fact that research by Desmond Ang (2023) has shown that counties that were visited by the traveling "road show" of the influential racist film *Birth of a Nation* on its five-year tour subsequently had spikes in lynchings and race riots. If substantial missing data made the lynching counts excessively noisy, Ang's results would be hard to account for.

Detailed results

Figure A2 shows Black life expectancy, White life expectancy, and the White–Black life expectancy gap by the 1860 percent enslaved. The top panel includes all states with an 1860

enslaved population of at least one percent; the bottom panel additional excludes Washington, DC, since it is an extreme outlier for White life expectancy among these states, in order to ensure that the overall relationship between White life expectancy and the proportion enslaved is not obscured.[2] The graphs show that, among states that had enslaved populations in 1860, although the relative size of this population varied tremendously, it has only a small relationship to Black lifespan today. States with larger enslaved populations have, on average, lower White lifespans today, but that relationship is driven very heavily by the high White lifespans in Washington, DC; when Washington, DC, is omitted, the difference in White lifespan between states with 10% and states with 50% enslaved populations in 1850 is about a single year. Accordingly, across the bulk of states that enslaved people in 1860, there is a fairly small negative relationship between the state-level 1860 percent enslaved and the White–Black lifespan gap.

Figure A3 shows lifespans by the number of Jim Crow laws enacted before 1950. Here, the inclusion or exclusion of both Washington, DC, and Louisiana—which enacted more than twice as many Jim Crow laws as most other Jim Crow states—heavily influence the results. In general, Black lifespans are *higher* in states that enacted more Jim Crow laws (though this aggregate relationship is suppressed when low-lifespan Louisiana is included). Meanwhile, the sharply contrasting White lifespans between Washington, DC, and Louisiana produce an overall negative relationship between Jim Crow laws and White lifespans, which masks an aggregate relationship in the other direction—states that enacted more Jim Crow laws tend to have higher White lifespans, although there is a lot of variation—when those states are excluded.

---

[2] To balance comparability across graphs with legibility, all graphs omitting Washington, DC, have a y-axis scale ranging from ages 70-80 for lifespans and 0-6 years for lifespan differences; all graphs that include Washington, DC, have the same lifespan range for Black life expectancy but ages 70-90 for White life expectancy, and 0-15 years for lifespan difference. In general, the case for omitting Washington, DC, is stronger for White lifespans (where Washington, DC, is an extreme outlier), and thus also for White–Black lifespan gaps, than for Black lifespans.

Accordingly, there is an overall negative relationship between Jim Crow laws and the White–Black lifespan gap, but its size is small when Washington, DC, and Louisiana are not driving it.

Figure A4 shows lifespans as a function of Black school quality. States whose segregated Black schools were worse had moderately better Black lifespans or, once Washington, DC, is omitted, moderately worse Black and White lifespans. Without Washington, DC, driving a large aggregate relationship between poor Black schools and low White lifespans, Black school quality is close to uncorrelated with the White–Black lifespan gap.

Figure A5 shows (in its top row) lifespans by the count of violent incidents from the Burnham-Nobles archive and (in its bottom row) lifespans by Baker's HRR index. Because the Burnham-Nobles measure captures the fewest states (and because which states it does or doesn't capture is more arbitrary than the other measures), to see whether the divergent results across measures reflect differences in the measure or the sample, the bottom row also shows results for the HRR, limited to the states included in the Burnham-Nobles archive (which are the states with the highest HRR values). The count of violent Jim Crow incidents has close to zero statistical relationship with Black lifespans and a moderate negative relationship with White lifespans, producing a moderate *negative* relationship with the White–Black lifespan gap. The HRR index results are different: states' greater combined intensity of slavery and Jim Crow has a small negative association with Black lifespan but has a small positive association with White lifespan, producing a moderate positive association with the White–Black lifespan gap. Surprisingly, this relationship with the White–Black lifespan gap is the same when limited to states in the Burnham-Nobles archive, even though the direction of the relationship with lifespans is reversed: both Black and White lifespans are lower in the states with the highest HRR values compared with states with more moderate HRR values. This reflects that both Black and White lifespans

have a somewhat curvilinear (concave) relationship with the HRR index, so limiting the sample to one segment of the HRR distribution changes the overall relationship to lifespan.

Finally, turning from states to counties, Figure A6 shows these outcomes in relation to county-level lynchings of Black Americans, with and without counties in which no lynching was identified (which may sometimes reflect a lack of newspaper coverage in the county for long stretches of time). Black lifespans are lower in counties with more Black lynchings—but White lifespans are even lower, so the White–Black difference is lower, too.

References to the Appendix

Ang, Desmond. 2023. "*The Birth of a Nation:* Media and Racial Hate." *American Economic Review* 113 (6): 1424–60. https://doi.org/10.1257/aer.20201867.

Bedingfield, Sid, and Kathy Roberts Forde. 2021. "Journalism and the World It Built." In *Journalism and Jim Crow*, edited by Sid Bedingfield and Kathy Roberts Forde, 1–28. White Supremacy and the Black Struggle for a New America. University of Illinois Press. http://www.jstor.org.ezp3.lib.umn.edu/stable/10.5406/j.ctv23r3fz8.5.

Brundage, W. Fitzhugh. 2021. "The Press and Lynching." In *Journalism and Jim Crow*, edited by Kathy Roberts Forde and Sid Bedingfield, 83–114. White Supremacy and the Black Struggle for a New America. University of Illinois Press. http://www.jstor.org/stable/10.5406/j.ctv23r3fz8.8.

Kelley, Blair LM. 2021. "Silencing a Generation." In *Journalism and Jim Crow*, edited by Kathy Roberts Forde and Sid Bedingfield, 285–304. White Supremacy and the Black Struggle for a New America. University of Illinois Press. http://www.jstor.org/stable/10.5406/j.ctv23r3fz8.16.

Perloff, Richard M. 2000. "The Press and Lynchings of African Americans." *Journal of Black Studies* 30 (3): 315–30. https://doi.org/10.1177/002193470003000303.

Roberts, Gene, and Hank Klibanoff. 2007. *The Race Beat: The Press, the Civil Rights Struggle, and the Awakening of a Nation*. 1st ed. New York: Knopf.

Seguin, Charles, and David Rigby. 2019. "National Crimes: A New National Data Set of Lynchings in the United States, 1883 to 1941." *Socius: Sociological Research for a Dynamic World* 5 (January): 237802311984178. https://doi.org/10.1177/2378023119841780.

The Civil Rights & Restorative Justice Project. n.d. "The Burnham-Nobles Digital Archive." Accessed October 1, 2022. https://www.crrjarchive.org/crrj.

APPENDIX TABLES

	**Difference between bivariate fitted-value lifespan outcome at racial subjugation measure's 75th vs. 25th percentile values**		
	**Black life expectancy**	**White life expectancy**	**White–Black life expectancy gap**
**1860 percent enslaved**	0.10	*-1.85*	*-1.95*
**1860 percent enslaved, Washington, DC excluded**	-0.19	-0.36	-0.17
**Jim Crow Laws passed before 1950**	0.20	*-0.89*	*-1.09*
**Jim Crow Laws, Washington, DC and Louisiana excluded**	0.32	0.26	-0.07
**Jim Crow school quality (reverse-coded)**	0.05	*-2.07*	*-2.11*
**Jim Crow school quality (reverse-coded), Washington, DC omitted**	-0.30	-0.37	-0.07
**Historical Racial Regimes index score**	-0.18	0.25	0.43
**Jim Crow archive-documented violent incidents**	-0.04	-0.38	-0.34
**Newspaper-reported Black lynchings (1883-1941), county-level**	-0.12	-0.06	-0.04

Table A1. Difference between lifespan fitted values, from bivariate linear regressions of the lifespan outcomes on the racial subjugation measure, at the 75th vs. 25th percentile of the racial subjugation measure. Table A1 repeats Table 1 with the addition of the two final rows, representing additional analyses. Lifespans are life expectancies at the state level from 2017-2019 or, for lynchings with Black victims, smoothed life expectancies at the county level from 2015-2019. The analysis of percent enslaved is limited to states which it exceeds 1%; the analysis of county lynching is limited to counties with at least one lynching. Italicized values are from regressions that are not preferred because they are heavily driven by very high white life expectancy in Washington, DC.

APPENDIX FIGURES

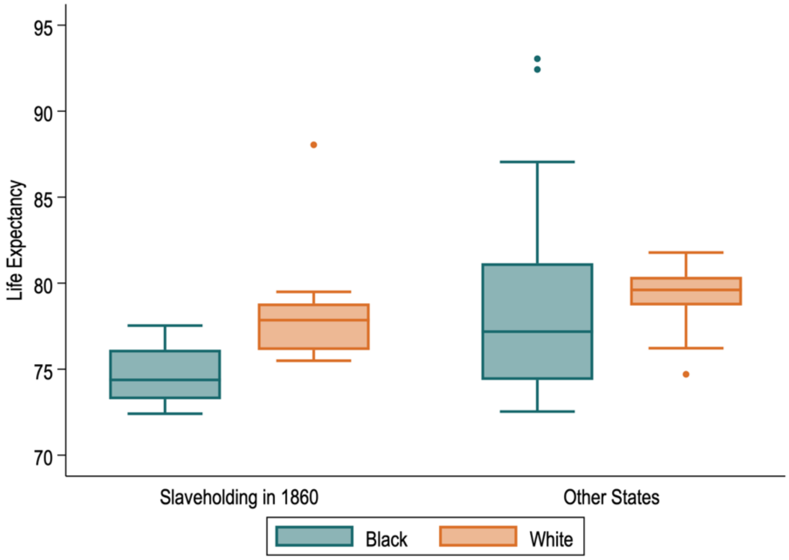

Figure A1. The distribution of state-level life expectancy (2017-2019) for non-Hispanic Black and non-Hispanic White populations in states that did vs. did not have enslaved populations in 1860. Life expectancy values that are clear outliers in the overall lifespan distribution should be interpreted cautiously, but do not substantially affect the median values reported in the text.

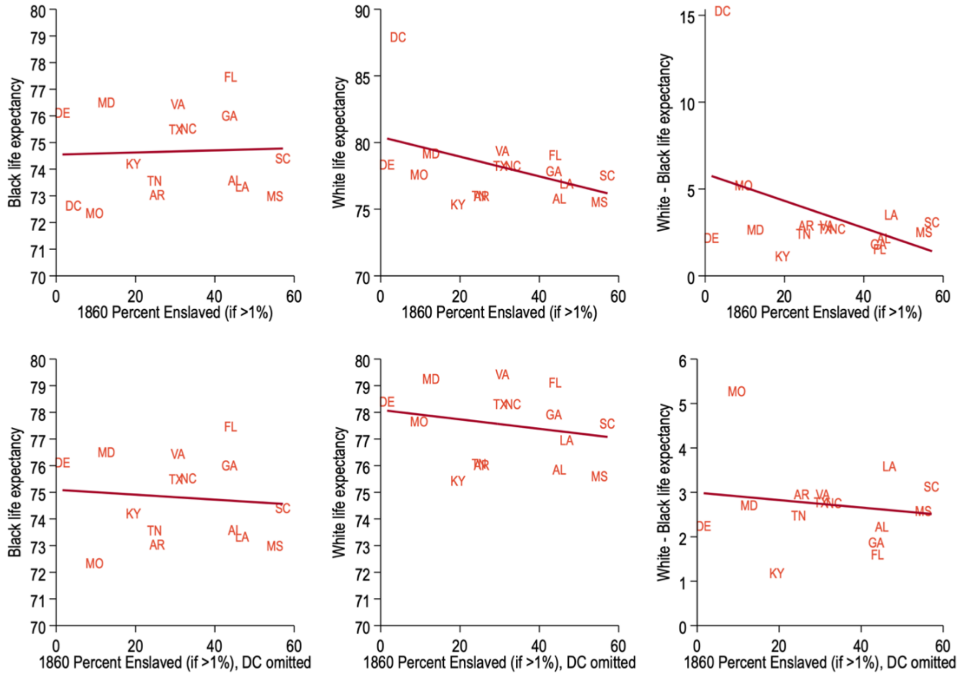

Figure A2. State-level lifespans (2017-2019) as a function of the 1860 percent enslaved.

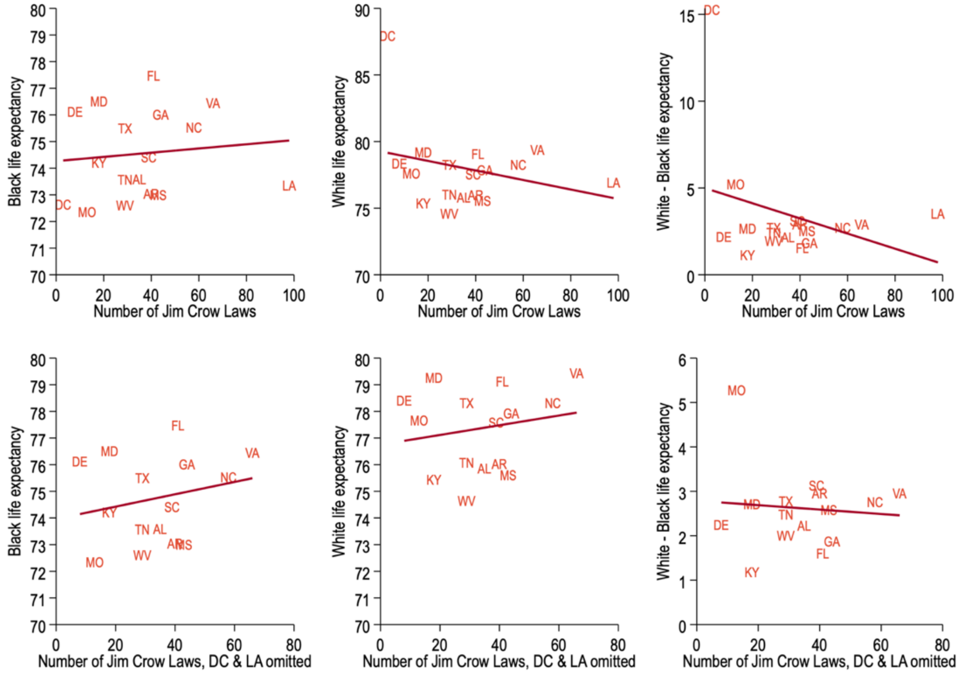

Figure A3. State-level lifespans (2017-2019) as a function of the number of Jim Crow laws passed before 1950.

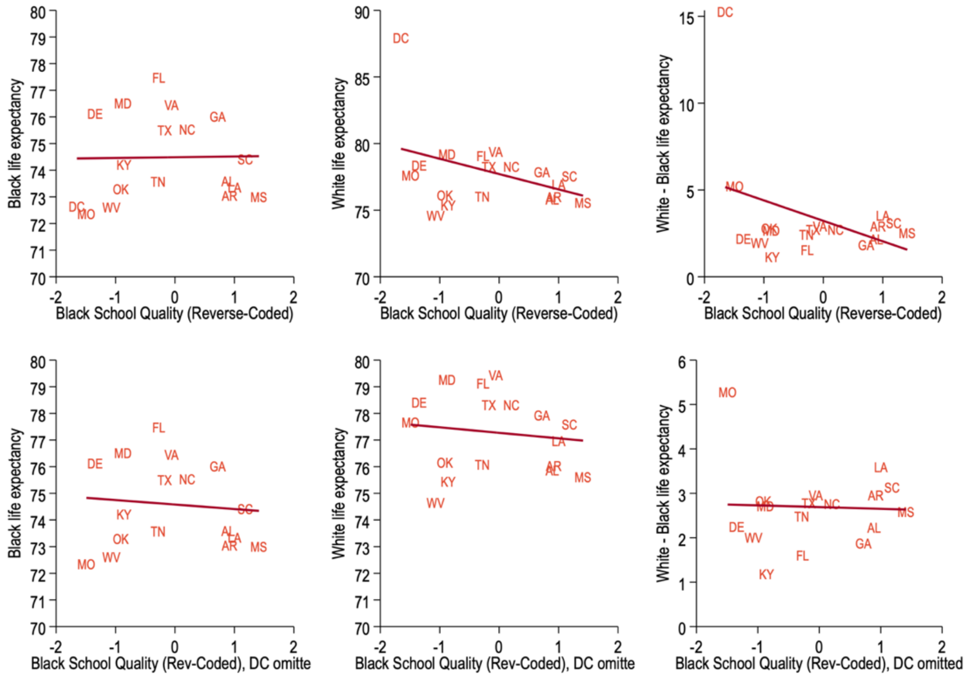

Figure A4. State-level lifespans (2017-2019) as a function of Jim Crow-era Black school quality (reverse-coded).

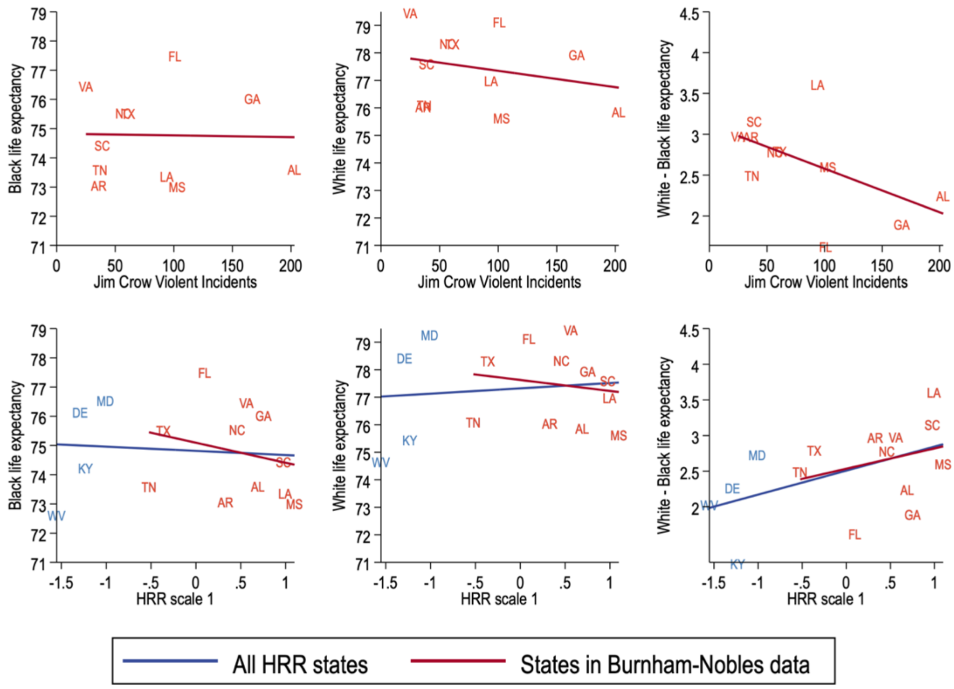

Figure A5. State-level lifespans (2017-2019) as a function of Jim Crow violent incidents (top row) and the Historic Racial Regime (bottom row), in total and on states with measures of Jim Crow violent incidents.

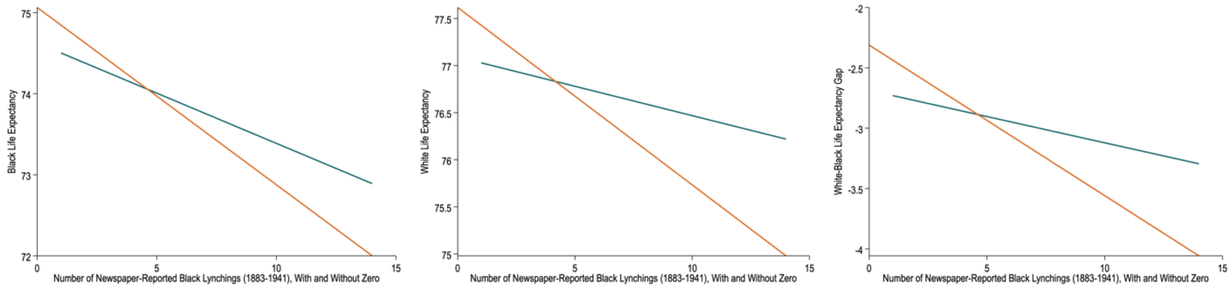

Figure A6. County-level lifespans (2015-2019) as a function of the count of county-level lynchings with Black victims (1883-1941), as reported in newspaper archives. In each panel, one fitted line includes, and one omits, counties with zero reported lynchings, which may reflect a true zero or may reflect a lack of newspaper coverage in the area during some of this time period.
